# *Odd-Paired* is Involved in Morphological Divergence of Snail-Feeding Beetles

**DOI:** 10.1093/molbev/msae110

**Published:** 2024-06-10

**Authors:** Junji Konuma, Tomochika Fujisawa, Tomoaki Nishiyama, Masahiro Kasahara, Tomoko F Shibata, Masafumi Nozawa, Shuji Shigenobu, Atsushi Toyoda, Mitsuyasu Hasebe, Teiji Sota

**Affiliations:** Department of Biology, Faculty of Science, Toho University, Funabashi, Chiba, Japan; Center for Data Science Education and Research, Shiga University, Hikone, Shiga, Japan; Research Center for Experimental Modeling of Human Disease, Kanazawa University, Ishikawa, Japan; Graduate School of Frontier Science, The University of Tokyo, Kashiwa, Chiba, Japan; National Institute for Basic Biology, Okazaki, Aichi, Japan; Department of Biological Sciences, Tokyo Metropolitan University, Hachioji, Tokyo, Japan; Research Center for Genomics and Bioinformatics, Tokyo Metropolitan University, Hachioji, Tokyo, Japan; National Institute for Basic Biology, Okazaki, Aichi, Japan; Department of Genomics and Evolutionary Biology, National Institute of Genetics, Mishima, Shizuoka, Japan; National Institute for Basic Biology, Okazaki, Aichi, Japan; Department of Basic Biology, The Graduate School for Advanced Studies (SOKENDAI), Okazaki, Aichi, Japan; Department of Zoology, Graduate School of Science, Kyoto University, Sakyo, Kyoto, Japan

**Keywords:** QTL mapping, RNAi, genome assembly, shape and size, opa, Zic gene family

## Abstract

Body shape and size diversity and their evolutionary rates correlate with species richness at the macroevolutionary scale. However, the molecular genetic mechanisms underlying the morphological diversification across related species are poorly understood. In beetles, which account for one-fourth of the known species, adaptation to different trophic niches through morphological diversification appears to have contributed to species radiation. Here, we explored the key genes for the morphological divergence of the slender to stout body shape related to divergent feeding methods on large to small snails within the genus *Carabus*. We show that the zinc-finger transcription factor encoded by *odd-paired* (*opa*) controls morphological variation in the snail-feeding ground beetle *Carabus blaptoides*. Specifically, *opa* was identified as the gene underlying the slender to stout morphological difference between subspecies through genetic mapping and functional analysis *via* gene knockdown. Further analyses revealed that changes in *opa cis*-regulatory sequences likely contributed to the differences in body shape and size between *C. blaptoides* subspecies. Among *opa cis*-regulatory sequences, single nucleotide polymorphisms on the transcription factor binding sites may be associated with the morphological differences between *C. blaptoides* subspecies. *opa* was highly conserved in a wide range of taxa, especially in beetles. Therefore, *opa* may play an important role in adaptive morphological divergence in beetles.

## Graphical abstract

**Graphical abstract:**
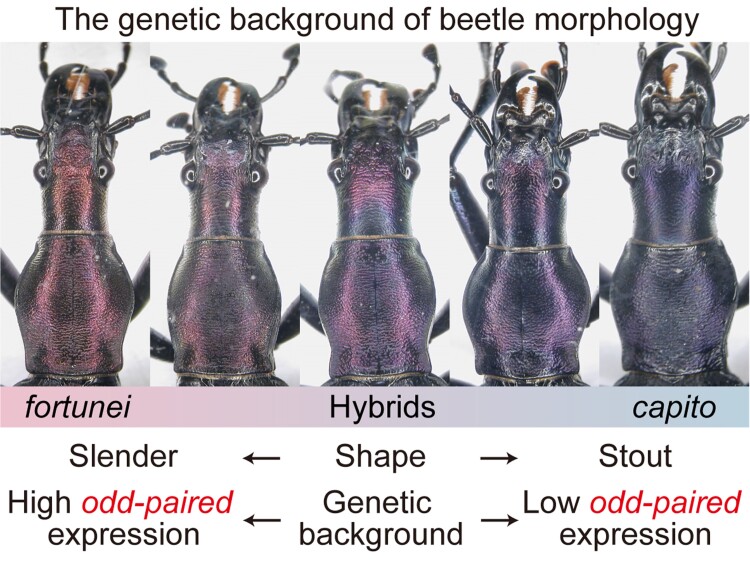

## Introduction

Comparative studies on body shape and size have revealed macroevolutionary patterns in morphological diversification (Friedman et al. 2019; Law 2021), including correlations between morphological evolutionary rates and species richness (Rabosky and Adams 2012), and have been used to comprehend morphological divergence in adaptive radiation (Harmon et al. 2010). Revealing the molecular basis of morphological diversification is essential for understanding the mechanisms underlying adaptive radiation. Toward this end, genome-wide association studies have identified candidate genes related to shape and size variations involved in adaptive diversification (Lamichhaney et al. 2015, 2016). In some organisms, genes involved in morphogenesis and other traits affect phenotypes mainly through *cis*-regulatory changes in their gene expression (Wray 2007; Carroll 2008; Wittkopp et al. 2008). However, the relative contribution of regulatory changes in gene expression to adaptive divergence remains poorly understood and an important focus of current genomic studies on parallel evolution and adaptive radiation (Stapley et al. 2010; Elmer and Meyer 2011; Berner and Salzburger 2015).

**Fig. 1.**
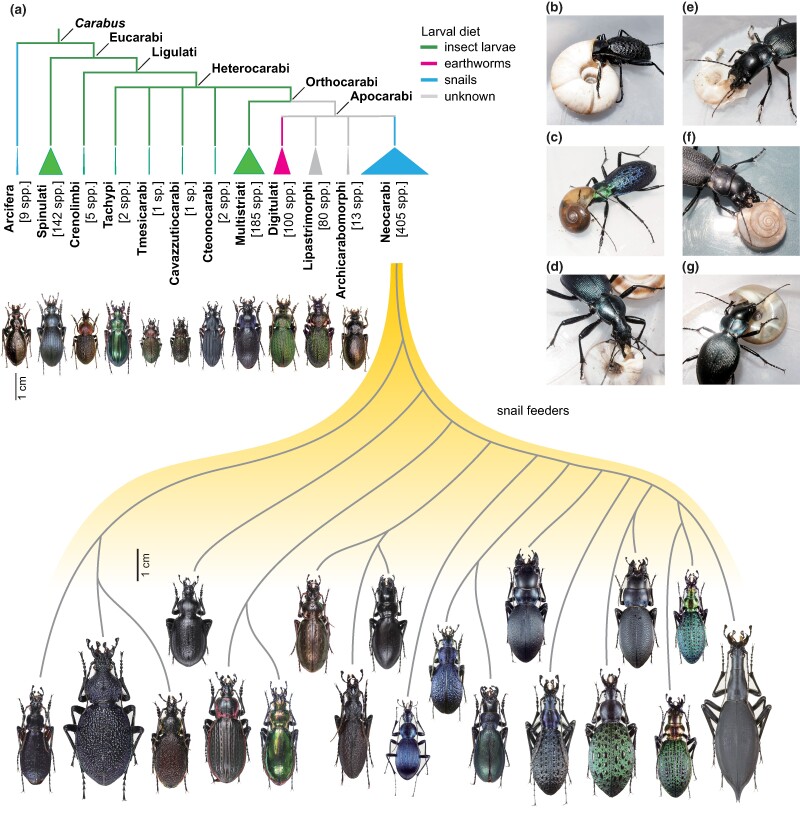
Adaptive radiation of snail-feeding *Carabus* beetles. a) Phylogenetic groups, species numbers, and diet types of the genus *Carabus* and representative species with phylogenetic relationships of the major snail-feeding group Neocarabi, based on previous reports (Deuve 2019; Sota 2022; Sota et al. 2022). b to g) Snail-feeding behavior of slender- (b to d) and stout-type (e to g) beetles in the Neocarabi group. Copyrights for all photographs and illustrations belong to T. Sota.

Beetles (Coleoptera) are the most species-rich insect group with 350,000 to 400,000 species, accounting for approximately one-fourth of all known species (Stork et al. 2015) and exhibiting remarkable morphological diversity in body shape and size. Morphological diversification of snail-feeding ground beetles of the genus *Carabus* (Carabidae: subfamily Carabinae) is an emerging model system used to investigate adaptive radiation in beetles. Snail-feeding carabid beetles evolved from ancestral carabid beetles feeding on insect larvae or earthworms and account for half of the diverse array of approximately 1,000 species in the genus *Carabus* (Sota 2022; Fig. 1a). Notably, the head/thoracic morphology of snail-feeding beetles has diverged into slender and stout forms (Sturani 1962; Ishikawa 1978), which are adapted to different feeding methods, i.e. shell-entry and shell-crushing, respectively [Vermeij 1979; DeWitt et al. 2000; Konuma and Chiba 2007; Konuma et al. 2013a (Fig. 1b to g; supplementary fig. S1c and d, Supplementary Material online)]. Local adaptation to shell sizes (Konuma et al. 2011) and character displacement (Akiyama et al. 2020) has led to adaptive radiation in snail-feeding carabid beetles. Previous quantitative genetic studies have shown that a small number of loci control slender to stout body shapes (Konuma et al. 2013b, 2014); however, the genes involved in these morphological differences remain unknown.

**Fig. 2.**
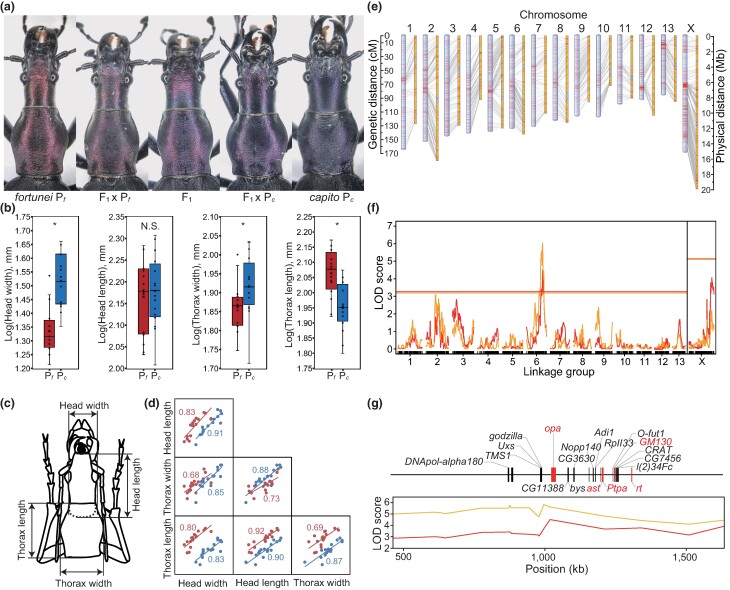
Morphology of slender and stout *C. blaptoides* subspecies and their hybrids, *C. b. fortunei* genome, and body-shape QTL location. a) Head–thorax morphology of *C. b. fortunei* (P*_f_*), *C. b. capito* (P*_c_)*, and their F_1_ and backcrossed individuals. The backcross between P*_f_* and F_1_ was used in the linkage and QTL mapping analyses. b) Box plots showing the morphological differences between P*_f_* (red, *n* = 16) and P*_c_* (blue, *n* = 16). The box plots show mean (cross), median (center line), first, and third quartiles (box limits), and 1.5 × interquartile range (whiskers). *, *P* < 0.05; N.S., *P* > 0.05; by general linear model; *F*_1,28_ = 70.65, *P* = 3.8 × 10^−9^ for head width; *F*_1,28_ = 6.04, *P* = 0.02 for thorax width; *F*_1,28_ = 21.98, *P* = 6.5 × 10^−5^ for thorax length; and *F*_1,28_ = 0.33, *P* = 0.57 for head length. c) Measured dimensions of a beetle head and thorax. The broken line represents the outline of the head base buried in the thorax. d) Scatter plots between the measured dimensions for P*_f_* (red) and P*_c_* (blue). Numerals represent correlation coefficients. e) Genetic map and corresponding assembled genome sequences of *C. b. fortunei*. The genetic map (left bars) was constructed *via* linkage mapping analysis using RAD markers (red lines) and consists of 14 linkage groups corresponding to the number of *C. blaptoides* chromosomes. The right bar in each linkage group shows corresponding genome sequences linked with multiple or single RAD markers (yellow and grey sections, respectively). f) LOD scores for the head width (red) and length (orange) using the thorax width as a covariate (see supplementary fig. S4, Supplementary Material online for the other morphological dimensions). Horizontal lines indicate LOD thresholds at the 5% significance level. g) Enlarged map of the QTL region (95% credible interval) on LG6, showing the LOD scores and the positions of DEGs (see supplementary table S3, Supplementary Material online for details). Five genes involved in gene ontology “developmental processes” are indicated by red letters and bars. The photographs and illustrations are copyrighted by J. Konuma.

In this study, we identified the genes controlling the body shape of the snail-feeding carabid beetles *Carabus blaptoides fortunei* and *C. b. capito* (Fig. 2a and supplementary fig. S1, Supplementary Material online) through de novo genome assembly, quantitative trait locus (QTL) mapping, gene expression analysis, RNA interference (RNAi), and resequencing of the two subspecies. Our findings suggest that the transcription factor gene *odd-paired* (*opa*) is responsible for the differences in body shape and size in *C. blaptoides*. *opa* is a pair-rule gene in *Drosophila* and a founding member of the zinc finger of the cerebellum (*Zic*) gene family (Hursh and Stultz 2018); consistent with our findings, *Zic* genes are the causal genes for head malformations in *Drosophila* (Lee et al. 2007) and humans (Brown et al. 1998; Grinberg et al. 2004; Twigg et al. 2015). Our study, therefore, highlights the potentially key role of conserved transcription factors in morphological divergence during beetle diversification.

## Results

### Genome Assembly

We assembled the genomic sequences of a *C. b. fortunei* male with Illumina paired-end short reads (insert size: 180 bp to 20 kb; supplementary table S1, Supplementary Material online) using the genome assembler Platanus (Kajitani et al. 2014). PacBio long reads from the same individual were used to close the gaps within the genomic scaffolds. The assembly consisted of 42,502 scaffolds totaling 188 Mb (supplementary table S2, Supplementary Material online), which was close to the genome size estimated using *k*-mer analysis (174 Mb). Using the total evidence approach for gene predictions with RNA-Seq data from the juvenile and adult stages of *C. b. fortunei*, we identified 16,189 protein-coding genes in the *C. blaptoides* genome and assigned these to the known genes of *Drosophila melanogaster*. To construct chromosome-level linkage groups, we applied restriction site-associated DNA sequencing (RAD-Seq) to 132 backcrossed individuals (BC1) of (*C. b. fortunei* × *C. b. capito*) × *C. b. fortunei* and performed linkage analysis; 1,533 RAD markers were grouped into 14 linkage groups (LGs), consistent with the chromosome number of *C. blaptoides* (Fig. 2e). The total length of the genetic map was 1,790 cM (marker density, 0.9/cM). Finally, we located 145 scaffolds on the genomic map, which had a total length of 156 Mb (83% of the assembled genome size).

### Trait-associated Gene Characterization

*C. b. capito* had a wider head and wider, shorter thorax than *C. b. fortunei* (Fig. 2b and c). In each subspecies, there were positive correlations between head and thorax widths and lengths (Fig. 2d). Because similar correlations were also observed in BC1 used in our QTL mapping (supplementary fig. S2, Supplementary Material online), we calculated the logarithm of odds (LOD) scores of one objective dimension using another as a covariate. In the genetic map, we identified a shared QTL associated with head width and length on LG6 using thorax width as a covariate (Fig. 2f; permutation test, *P* = 3.2 × 10^−3^ for head width; *P* = 1.1 × 10^−4^ for head length). Beetles heterozygous for this QTL had wider and longer heads than those homozygous for the *C. b. fortunei* allele (supplementary fig. S3, Supplementary Material online). Since both head width and length were correlated with thorax width, the LOD scores of thorax width also supported the QTL detected using head width or head length as a covariate (permutation test, *P* = 0.046 and *P* = 9.9 × 10^−4^, respectively; supplementary fig. S4, Supplementary Material online). No significant differences were found in thorax width and length between the genotypes on the QTL (supplementary fig. S3, Supplementary Material online). Thus, the QTL affected the head rather than the thorax; the *C. b. capito* allele increased the head width and length, whereas the *C. b. fortunei* allele reduced them. This QTL explained 5.63% and 5.59% of the head width and length variation, respectively.

We detected 108 genes within the 95% credible interval of the QTL and performed comparative gene expression analysis using transcriptome data from the prepupae of *C. b. fortunei* and *C. b. capito*, assuming that genes exhibiting differential expression between the subspecies and located in the QTL region were responsible for the shape difference. Among the 108 genes, we identified 23 differentially expressed genes [DEGs (supplementary table S3, Supplementary Material online)], of which five were involved in the gene ontology category “developmental process” (Gene Ontology Consortium 2004): *opa*, *asteroid* (*ast*), *phosphotyrosyl phosphatase activator* (*Ptpa*), *golgi matrix protein 130 kD* (*GM130*), and *rotated abdomen* (*rt*). Of these, *opa* was closest to the QTL peak (Fig. 2g). *C. b. fortunei opa* (hereafter *Cbf-opa*) was more highly expressed than *C. b. capito opa* (hereafter *Cbc*-*opa*) in both the heads [false discovery rate (FDR)-adjusted *P* = 5.8 × 10^−3^] and thoraxes (FDR-adjusted *P* = 4.2 × 10^−2^). *ast*, *GM130*, and *rt* are associated with the development of wing veins (Dworkin and Gibson 2006), neuronal dendrites (Liu, Mei, et al. 2017), and abdominal asymmetry (Martín-Blanco and García-Bellido 1996), respectively. *Ptpa* is not associated with anatomical structure development, but rather with the regulation of neuroblast asymmetric divisions (Zhang et al. 2016). Other DEGs in the QTL were involved in “metabolic processes,” “cellular processes,” or were undetermined with respect to gene ontology. Therefore, we considered *opa* as the sole candidate gene for regulating head morphology.

### Gene Function

**Fig. 3.**
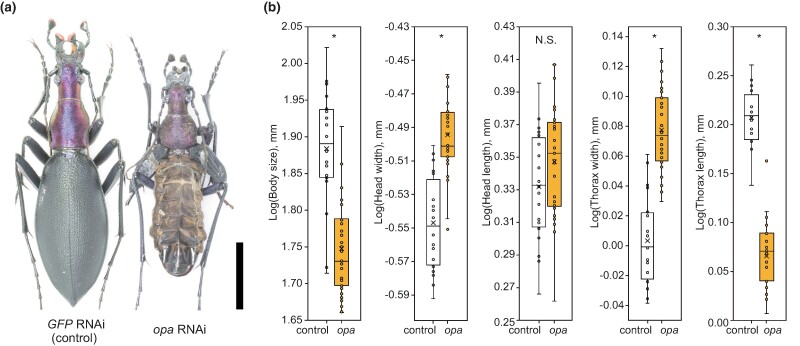
RNAi-mediated *opa* knockdown. a) Left: *GFP* dsRNA-injected *C. b. fortunei* (control). Right: *opa* dsRNA-injected *C. b. fortunei*. Scale bar, 10 mm. b) Box plots showing the morphological differences between the controls (white, *n* = 20) and *opa* dsRNA-injected beetles (yellow, *n* = 32). The box plots show mean (cross), median (center line), first and third quartiles (box limits), and 1.5 × interquartile range (whiskers). Body size is the geometric mean of all the measurements. *Analysis of variance (ANOVA), *F*_1,50_ = 46.04, *P* = 1.3 × 10^−8^ for body size. The four morphological dimensions were size-corrected using Burnaby's procedure (Burnaby 1966); *ANOVA, *F*_1,50_ = 46.54, *P* = 1.1 × 10^−8^ for head width; *F*_1,50_ = 88.68, *P* = 1.2 × 10^−12^ for thorax width; *F*_1,50_ = 247.74, *P* = 5.2 × 10^−21^ for thorax length, N.S. (not significant); and *F*_1,50_ = 2.57, *P* = 0.12 for head length. The photographs and illustrations are copyrighted by J. Konuma.

Since slender *C. b. fortunei* had higher expression of *opa* in the prepupal stage than stout *C. b. capito*, we expected that *C. b. fortunei* with knockdown of *opa* expression would develop a stout morphology similar to that of *C. b. capito*. To test this possibility, we conducted a systemic larval RNAi experiment using the slender subspecies *C. b. fortunei*. Among the final instar larvae injected with *opa* double-stranded RNA (dsRNA), 50% emerged as adults, whereas the remaining individuals died during the pupal and prepupal stages (supplementary table S4, Supplementary Material online). Body size and shape differences were observed in the emerging adults between *opa* dsRNA-injected and control groups (Fig. 3). *opa* dsRNA-injected beetles were smaller with wider heads and wider, shorter thoraxes, comparable to those in *C. b. capito*. They also had unextended elytra (Fig. 3a), which indicated that the expression of *opa* was essential for elytral formation.

### *cis*-Regulatory Sequences

**Fig. 4.**
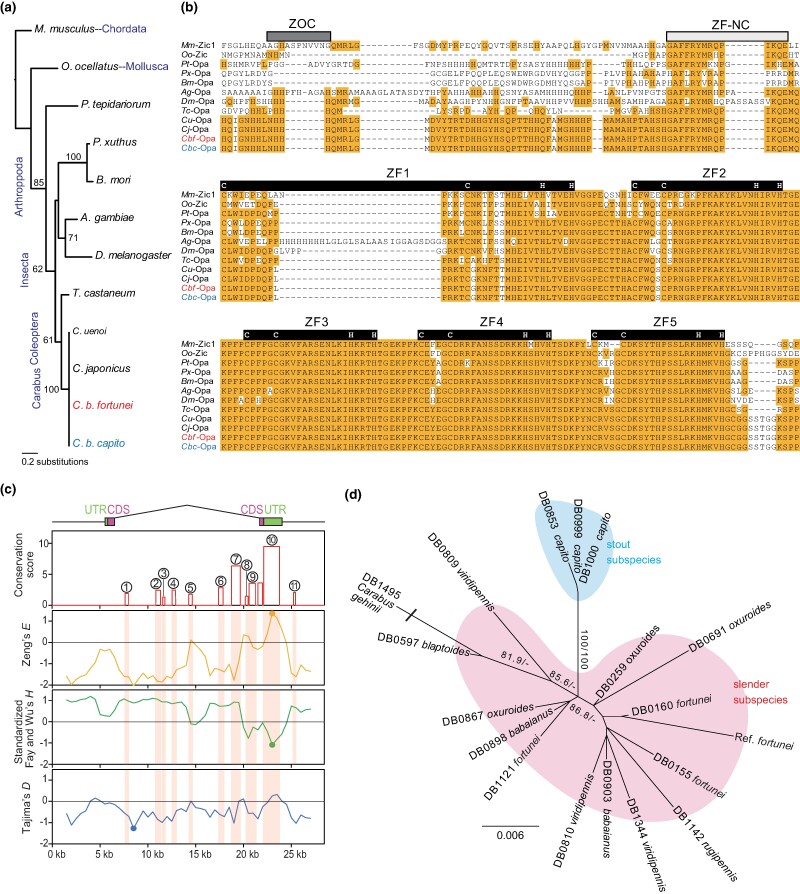
Amino-acid and nucleotide sequence comparisons for Opa. a) Phylogenetic relationships among Opa and Zic amino-acid sequences of 12 taxa in Chordata, Mollusca, and Arthropoda. b) Aligned amino-acid sequences encompassing five zinc-finger (ZF) domains (ZF1–5; black bars), Zic–Opa conserved (ZOC) domain (dark grey bar), and the ZF–NC domain positioned N-terminal to ZF1 (light grey bar). Letters with orange backgrounds indicate consensus sequences. White letters in the black bars indicate the positions of C_2_H_2_ motifs in the ZF domains (see supplementary fig. S5, Supplementary Material online for the entire Zic/Opa sequences). Taxon names and codes: house mouse, *Mus musculus* (*Mm*-Zic1); octopus, *Octopus ocellatus* (*Oo*-Zic); spider, *Parasteatoda tepidariorum* (*Pt*-Opa); swallowtail butterfly, *Papilio xuthus* (*Px*-Opa); silkworm moth, *Bombyx mori* (*Bm*-Opa); mosquito, *Anopheles gambiae* (*Ag*-Opa); fruit fly, *Drosophila melanogaster* (*Dm*-Opa); red flour beetle, *Tribolium castaneum* (*Tc*-Opa); *Carabus uenoi* (*Cu*-Opa); *C. japonicus* (*Cj*-Opa); *C. b. fortunei* (*Cbf*-Opa; red text); and *C. b. capito* (*Cbc*-Opa; blue text). c) Eleven conserved non-coding sequences (CNSs) in *opa* as identified using the conservation score. From top: conservation score; Zeng's *E*-value; standardized Fay and Wu's *H*-value; Tajima's *D*-value. The red-shaded areas are the CNS regions indicated by the circled numbers. The highest *E* peak and the lowest *H* and *D* peaks are represented with dots. d) Maximum-likelihood tree for the partial sequences of the CNS10 region (defined in Methods) among seven *C. blaptoides* subspecies. Node support values are SH-aLRT and the ultrafast bootstrap percentages (shown when > 80% and > 95%, respectively).

Evaluation of sequence differences in the *opa* gene region between *C. b. fortunei* and *C. b. capito* using resequencing data (*n* = 22 for each subspecies) revealed only a single synonymous substitution; the amino-acid sequences of the open reading frame were identical between *Cbf*-Opa and *Cbc*-Opa (supplementary fig. S5, Supplementary Material online). In the functionally important domains of *opa* [ZF, ZOC, and ZF–NC (Hursh and Stultz 2018)], the *Cbf*-Opa/*Cbc*-Opa amino-acid sequences were identical to the Opa sequences of *Carabus uenoi* and *C. japonicus* (Komurai et al. 2017; Fujisawa et al. 2019), which are congeneric earthworm feeders distantly related to *C. blaptoides* [*Cu*-Opa and *Cj*-Opa (Fig. 4b)]. Furthermore, the sequences of these domains were almost identical to those of *Tribolium castaneum* (*Tc*-Opa).

We assumed that a mutation in the non-coding sequence would result in the *opa* expression difference *via cis*-regulatory changes (Wray 2007; Carroll 2008; Wittkopp et al. 2008). *cis*-regulatory elements tend to be conserved between related species because they are functionally important and subject to negative selection (Zhang and Gerstein 2003). Therefore, conserved non-coding sequences [CNSs (Frazer et al. 2004)] are candidate *cis*-regulatory elements. To identify the CNSs, we compared *opa* and its flanking sequences between the two *C. blaptoides* subspecies and the two *Carabus* species, *C. japonicus* and *C. uenoi* (Komurai et al. 2017; Fujisawa et al. 2019), and found 11 CNSs more highly conserved than expected under neutral evolution (Fig. 4c). Of these, CNS10, which encompasses most of the 3′ untranslated region (UTR), had the highest conservation score.

Next, we attempted to detect evidence of natural selection that might have acted on the *opa* sequence, using a population genetic approach. Since a peak shift from a slender to a stout body shape appeared to have occurred after the ancestor of *C. b. capito* colonized Sado Island (Konuma et al. 2013a), it is likely that a new stout-type mutation occurred in the ancestral beetles on Sado Island, and positive selection increased the stout-type frequency. We hypothesized that a selective sweep (Smith and Haigh 1974) occurred in the *opa* sequence of *C. b. capito*. To identify a candidate location for the selective sweep, we examined the peak of three statistics that indicated a footprint of positive selection: Zeng's *E* value (Zeng et al. 2006), standardized Fay and Wu's *H*-value (Fay and Wu 2000; Zeng et al. 2006), and Tajima's *D*-value (Tajima 1989), in the sequence of *Cbc*-*opa* (Fig. 4c). Zeng's *E* and standardized Fay and Wu's *H*-values were the highest and lowest, respectively, in CNS10. Compared with the simulated values under neutral evolution, the observed *E* peak was higher (one-tailed test, *P*[Sim ≤ Obs] = 0.9971) and the standardized Fay and Wu's *H* peak was lower (one-tailed test, *P*[Sim ≤ Obs] = 0.0061). These results indicate that some non-neutral evolution, presumably a selective sweep, occurred in CNS10 of *C. b. capito*. However, Tajima's *D* was the lowest in the intron region, not in the CNS, and was lower than the simulation value (one-tailed test, *P*[Sim ≤ Obs] = 0.0000). We also compared the partial sequences of the CNS10 region among the seven *C. blaptoides* subspecies. All slender subspecies shared CNS10 sequences similar to those of *C. b. fortunei*, whereas the stout subspecies *C. b. capito* had specific sequences of CNS10 that differed from that of all slender subspecies (Fig. 4d). This result supports the association of CNS10 with the slender to stout morphology of *C. blaptoides* and its likely identity as the causative *cis*-regulatory element.

Since it was suggested that base substitutions within this CNS10 region may be responsible for the differential gene expression, we examined potential transcription factor binding sites within this region using JASPAR (https://jaspar.elixir.no/). We found 233 sites of 5 to 12 bp to which 112 insect transcription factors could bind within the CNS10 region of *C. b. fortunei* (supplementary fig. S6, Supplementary Material online). Eleven SNPs for *C. b. capito* were found in 11 of the 233 binding sites. These SNPs may be responsible for the morphological differences between *C. b. capito* and *C. b. fortunei*. We also analyzed selective sweeps of the entire QTL region to check if the footprint of natural selection could be detected in other DEGs. We found high Zeng's *E* (supplementary fig. S7, Supplementary Material online) and low standardized Fay and Wu's *H* (supplementary fig. S8, Supplementary Material online) for *DNApol-alpha180* and *Uxs* as well as low Tajima's *D* (supplementary fig. S9, Supplementary Material online) for *bys*, *Nopp140*, *Adi1*, *Rpll33*, *Ptpa*, *O-fut1*, *CRAT*, CG7456, and *rt*. These genes may also be involved in the morphological differences in *C. blaptoides*.

## Discussion

In this study, we generated a de novo genome assembly of *C. blaptoides* using Illumina short reads and PacBio long reads, located the assembled sequences on a high-density genetic map, and reconstructed the 13 autosomes and the X-chromosome of *C. blaptoides*. In this chromosome-level genome, we identified a QTL associated with slender to stout morphological divergence. Among the DEGs in this QTL, we determined *opa* as a candidate gene associated with beetle morphology. We evaluated the expression levels of *opa* during the prepupal stage and found that *opa* expression was higher in the slender subspecies than in the stout subspecies. RNAi-mediated gene knockdown revealed that suppression of *opa* expression caused body shape change to a stouter form in the slender subspecies. Based on these results, we conclude that *opa* is the causal gene for the slender to stout morphological differences in *C. blaptoides*.

*C. b. fortunei* treated with *opa* RNAi showed some defects in adult eclosion, notably, a failure of elytral extension and reduction in body size. Defects caused by *opa* knockdown in adult eclosion have also been observed in *T. castaneum* (Linz and Tomoyasu 2015) and *D*. *melanogaster* (Simon et al. 2019). These results suggest that *opa* is likely a key gene for normal adult eclosion and affects body size and shape through its effects on the extension of body parts. The involvement of *opa* in controlling body size was also suggested by a QTL mapping study for body size differences among populations in *C. japonicus*, which is an earthworm-feeding species that does not exhibit noticeable body-shape variation (Komurai et al. 2017). Although *opa* was not listed among the candidate genes for body size variation in *C. japonicus*, we reviewed the annotated genes and found that *opa* was located in the body size QTL (supplementary table S5, Supplementary Material online). Thus, *opa* may be involved in body size variations in *Carabus* beetles.

*opa* is the *Drosophila* homolog of the *Zic* gene family, a family of transcription factors involved in metazoan morphogenesis (Layden et al. 2010). Zic/Opa proteins are well conserved in bilaterians such as Chordata, Mollusca, and Arthropoda [Aruga et al. 2006 (Fig. 4a)]. Human *ZIC* genes include the causal genes of head malformations such as holoprosencephaly (Brown et al. 1998), Dandy–Walker malformation (Grinberg et al. 2004), and craniosynostosis (Twigg et al. 2015). In *Drosophila*, Opa regulates the transcription of the *Drosophila* homolog of the *BMP* gene family, *decapentaplegic* (*dpp*; Sen et al. 2010), and is crucial for head morphogenesis (Lee et al. 2007). This study shows that *opa* plays an important role in beetle head morphogenesis. Thus, *opa* may affect head morphology in various insects.

Although the exact sequence differences responsible for the differential expression of *opa* remain to be determined, the results of the conservation score, Zeng's *E*, and the standardized Fay and Wu's *H* suggested that the region mainly consisting of the 3′ UTR is likely responsible for the main differences in subspecies expression. However, because Tajima's *D* suggests a footprint of positive selection in the intron, multiple differences may contribute additively. Our phylogenetic results for the *opa* 3′ UTR region imply that the ancestor of *C. b. capito* was a slender-type beetle and a stout-type mutation occurred and became fixed after colonization on the isolated Sado Island.

The function of *opa* in regulating *C. blaptoides* subspecific morphology requires further study. Our RNAi experiments had limited success in reproducing converse subspecific morphologies while affording high mortality and defects in the development of other body parts (e.g. elytra). The high mortality and defects could be attributed to the systemic application of dsRNA, which might have affected other important functions of *opa*. In addition, other genes may affect the subspecific morphological differences, as we found a marginally insignificant head length QTL on LG2 (Fig. 2f). A larger mapping population than that included in the present study would be required for detecting QTLs with less pronounced effects. RNAi experiments should also encompass other DEGs in the QTL, especially those with footprints of natural selection.

Morphological diversification in the shape and size of snail-feeding *Carabus* beetles may have occurred through local adaptation in response to different prey sizes (Konuma et al. 2011) along with character displacement (Akiyama et al. 2020). This kind of morphological diversification has occurred repeatedly and contributed to the diversification of snail-feeding *Carabus* species. Our results imply that this diversification may have been facilitated by *cis*-regulatory changes in the transcription factor *opa*, a member of *Zic* gene family. We propose that *opa* is a developmental gene that plays key roles in the adaptive divergence of body shape and size and contributes to beetle species diversity. An extended survey of responsible genes for body shape and size in various beetle taxa is therefore also warranted.

## Materials and Methods

### Organisms

*Carabus blaptoides* are snail-feeding carabid beetles endemic to the Japanese islands and are classified into eight subspecies exhibiting slender to stout head–thoracic morphology (Konuma et al. 2011). We selected the slender subspecies *C. b. fortunei* from Awashima Island (38.4596°N, 139.2403°E) and the stout subspecies *C. b. capito* from Sado Island (38.2181°N, 138.4105°E) for our genomic study of morphological divergence (Konuma et al. 2013b, 2014). These subspecies are genetically close to each other (Su et al. 1998), consistent with the small geographic distance of the islands. We used *C. b. fortunei* for whole-genome sequencing because it was derived from an isolated small island (9 km^2^), suggesting that the genome heterozygosity might be relatively low.

To examine morphological differences between *C. b. capito* and *C. b. fortunei*, both subspecies were spawned and reared to adults under identical environmental conditions in the laboratory (*n* = 16 for each subspecies). The width and length of the adult head and thorax were measured using a digital caliper [Mitutoyo Corp., Kawasaki, Japan (supplementary table S6, Supplementary Material online)] and then analyzed using a general linear model with log-transformed measurements as the response variable and subspecies and sex as explanatory variables. Pearson's product-moment correlation coefficients were calculated for the log-transformed head and thorax width and length, respectively.

### DNA Extraction and Sequencing

Total genomic DNA was extracted from the testes and muscle tissues of three *C. b. fortunei* males, produced from a single wild-caught female, using the DNeasy Blood & Tissue Kit (Qiagen, Hilden, Germany). The quality of the extracted DNA was confirmed using agarose gel electrophoresis. Two paired-end (PE) libraries (insert sizes: 180 and 500 bp) were prepared with 5 μg DNA from the firstborn among the three males using the Illumina TruSeq DNA Sample Preparation v2 Kit and were sequenced *via* 101-bp reads using the HiSeq 2000 (Illumina, San Diego, CA, USA). A PacBio library (insert size, 6 kb) was prepared using 6 μg DNA from the same male and sequenced on a PacBio RS (Pacific Bioscience, Menlo Park, CA, USA). For scaffolding contigs, Illumina mate-pair (MP) libraries (insert size: 2.5, 3.5, 4.5, 7.0, and 11 kb) were prepared using approximately 4 μg DNA from another male and sequenced using 101 bp reads on the HiSeq 2500 (Illumina). Genomic DNA from the remaining male was used for additional MP sequencing of long inserts (20 kb). All MP libraries were prepared using the Nextera Mate-Pair Sample Preparation Kit (Illumina).

### Genome Assembly

The PE reads were preprocessed to trim the Illumina adapter sequences using Trimmomatic v0.32 (Bolger et al. 2014) and assembled using Platanus v1.2.1 (Kajitani et al. 2014). The MP reads were preprocessed using NextClip v1.2 and classified into categories A, B, C, and D according to adapter detection status (Leggett et al. 2014). Using A, B, and C reads of the 20-kb MP reads and all A, B, C, and D reads of other MP reads, we scaffolded the assembled reads using Platanus v1.2.1. The PacBio long reads were preprocessed for error correction using Sprai v0.9.9.2 (https://kasahara-lab.github.io/sprai_doc/) and applied for gap closing of the assembled genome using PBJelly v15.2.20 (English et al. 2012). Ambiguous bases (N) were excluded from the seven scaffolds starting or ending with N, and three scaffolds with fewer than 100 bases were excluded. Genome size was estimated *via k*-mer analysis using JellyFish v2.0 (Marçais and Kingsford 2011) for comparison with the total length of the scaffolds in the assembled genome. To assess the quality of the genome assembly, we calculated a benchmarking universal single-copy orthologue (BUSCO) score using BUSCO v5.1.3 (Seppey et al. 2019), in which endopterygota_odb10 was used as the lineage dataset.

### Genotyping With RAD-sequencing

We used 132 backcrossed individuals obtained from the cross (*C. b. capito* female × *C. b. fortunei* male) females × *C. b. fortunei* male, produced in a previous study (Konuma et al. 2014), for our new linkage and QTL analyses. The genomic DNA of the backcrossed individuals and their grandparents was extracted using the DNeasy Blood & Tissue Kit; DNA quality was verified using a Bioanalyzer (Agilent Technologies, Santa Clara, CA, USA). The DNA was applied to construct RAD-sequencing libraries (Etter et al. 2011). *Pst*I was used as the restriction enzyme, and the samples were barcoded with 5-bp sequences. The libraries were sequenced with 101-bp single-end (SE) reads using the HiSeq 2000. Sequenced reads were preprocessed using Trimmomatic v0.32 and genotyped using Stacks v1.37 (Catchen et al. 2011). The reads, sorted by individual, were mapped on the assembled genome using Bowtie2 v2.2.9 (Langmead and Salzberg 2012), and SNPs were identified using “pstacks.” A catalog describing a set of all possible alleles expected in the backcrossed individuals was constructed by comparing grandparental SNPs using “cstacks.” The alleles of the backcrossed individuals were examined in the catalog using “sstacks.” The genotypes of all pedigrees were determined using “genotypes” with the parameter -t “BC1,” which output the homozygous and heterozygous genotypes as “b” and “h”, respectively. Parameter option -c, which enables data correction based on coverage depth, was also used. The loci for which the grandparental genotype was “aa × bb” were used as RAD markers in the subsequent linkage analysis.

### Genetic Linkage Map

Linkage analysis of the RAD markers was conducted using JoinMap v4.1 (Kyazma, Wageningen, The Netherlands). RAD markers that followed the expected segregation ratio (1:1) were filtered using chi-square tests at a significance level of *P* = 0.05. The filtered markers were grouped into LGs based on the test for independence with an LOD score threshold of 10. Markers were ordered within the LGs using a regression-mapping algorithm. The best marker positions were searched using goodness-of-fit tests in the first and second rounds, between which a ripple command was executed. The map distances were calculated using the Kosambi mapping function. This species has XY sex chromosomes, and the male genotypes are hemizygous for the X-chromosome. Thus, we could identify LGs in which males are only homozygotic and females are both homozygotic and heterozygotic as representing X chromosomes. To identify the X-chromosome, we additionally applied “genotypes” in Stacks with the parameter -t “F2' and output “a” and “b” for homozygotic loci and “h” for heterozygotic loci. Linkage groups on X chromosomes were assumed to have male genotypes “a” or “b” and female genotypes “a,” “b,” or “h.”

### Super-scaffolding

We located the scaffolds with RAD markers on a genetic map constructed using linkage analysis. Some of the scaffolds were split into two or three parts and mapped onto different LGs. These scaffolds likely contained contigs erroneously connected *via* 20-kb MP reads with low coverage depths. We calculated the coverage depths of the genomic regions in the scaffolds that contained possible erroneous connections *via* 20-kb MP reads using DepthHist (https://github.com/tomoakin/DepthHist). If the coverage depth of the genomic portion including the 20-kb MP reads was the lowest in the scaffold, we judged the connection to be incorrect and negated it.

### RNA-Seq for Gene Prediction

We obtained 12 *C. b. fortunei* samples from eight different developmental stages: an embryo, a first-instar larva, a second-instar larva, a prepupa, a pair of male and female pupae, a pair of male and female adults immediately after eclosion, a pair of male and female adults 3 h after eclosion, and a pair of male and female adults 1 wk after eclosion. Whole bodies were fixed in RNAlater solution (Thermo Fisher Scientific, Waltham, MA, USA) and preserved at −30 °C until RNA extraction. Total RNA was extracted from each sample using the RNeasy Mini Kit (Qiagen). RNA concentration was measured using the Qubit RNA BR Assay Kit (Thermo Fisher Scientific), and the RNA integrity number (RIN) was obtained using TapeStation 2200 (Agilent Technologies). The cutoff RIN used for the samples was 8. RNA-Seq libraries for 101-bp PE reads were prepared using the TruSeq Stranded mRNA Sample Preparation Guide (Illumina), evaluated for quality using a Bioanalyzer, and sequenced at 1201× coverage on the HiSeq 2500.

### Gene Prediction

A custom repeat library for the *C. blaptoides* assembly was constructed using RepeatScout (Price et al. 2005) and shared repeats for Hexapoda were downloaded from Repbase (https://www.girinst.org/repbase/). Then, repeats on the scaffolds were identified and masked using RepeatMasker (https://www.repeatmasker.org/) based on the two libraries. The mRNA reads from the eight developmental stages were mapped onto the scaffolds using GSNAP (Wu and Nacu 2010), and the exon–intron structure was assembled using Cufflinks (Trapnell et al. 2010) for each stage before the results of multiple stages were merged using CuffMerge. Multiple ab initio prediction algorithms were run using intron hints generated from transcriptome assembly. Specifically, Augustus (Stanke and Waack 2003) was run using the *Tribolium castaneum* model (which is based on the most well-annotated Coleoptera genome) and intron hints. GeneMark-ET (Lomsadze et al. 2014) was run by incorporating a semi-supervised training algorithm together with intron hints. Multiple pieces of evidence from the transcriptome assembly and the two ab initio predictions were integrated using EvidenceModeler (Haas et al. 2008), with the evidence weights set to 1 for ab initio predictions and 10 for transcriptome evidence.

### QTL Mapping

We used morphological data of 82 adult beetles (41 males and 41 females) obtained from a previous study [Konuma et al. 2014 (supplementary table S7, Supplementary Material online)]. The four morphological dimensions (head width, head length, thorax width, and thorax length) were log-transformed, and their association with the RAD-Seq genotypes was examined because these dimensions are phenotypic values that determine the snail-feeding success rate (Konuma et al. 2013a). We searched for QTLs associated with morphological dimensions using R/qtl v1.41-6 (Broman and Sen 2009). Prior to the QTL analyses, genotyping errors were checked using the “calc.errorld” function, which compared genotypes between closely located markers to detect erroneous genotypes. Genotypes with error LOD scores > 3 were considered as missing data. The “jittermap” function was also used to slightly separate the positions of overlapping markers. LOD scores were calculated on a dense grid of positions along the linkage map using the “scanone” function *via* a multiple imputation method. However, the correlation between the four morphological dimensions (Konuma et al. 2013b, 2014) renders QTL mapping difficult. To resolve this problem, we calculated the LOD scores of one objective dimension using another as a covariate. For example, the LOD scores of head width were calculated in three models in which head length, thorax width, and thorax length were used as covariates. A permutation test with 10,000 permutated data sets was conducted to determine the genome-wide LOD threshold at the 5% significance level for autosomes. A permutation test with 97,053 permutated datasets was conducted for the X-chromosome. The morphological differences between genotypes on the QTL were tested at a 5% significance level using analysis of variance (ANOVA). Bayesian credible intervals of QTL location were estimated using the “bayesint” function. The proportion of phenotypic variance explained by each QTL was estimated using the “fitqtl” function.

### RNA-Seq for Gene Expression Analysis

Morphological differences between *C. b. fortunei* and *C. b. capito* are observed in pupae but not in prepupae. Therefore, we assumed that the morphological differences between the two subspecies are caused by differences in gene expression in the prepupa. The head and thoracic parts of three laboratory-reared prepupae (second-instar larvae) of *C. b. fortunei* and *C. b. capito* were separately fixed in RNAlater solution. Total RNA was isolated from individual head or thorax samples, and RNA-Seq libraries were prepared as described above. SE sequencing was conducted at 89× coverage on the HiSeq 2500. The sequenced reads were preprocessed using Trimmomatic v0.32 and aligned to the *C. b. fortunei* reference genome using TopHat v2.1.1 (Trapnell et al. 2012) with the gene prediction model option. We counted the reads and compared the count data between *C. b. capito* and *C. b. fortunei* using Cufflinks v2.2.1 (Trapnell et al. 2012). Fragments per kilobase of exon per million reads mapped (FPKM) were summed in transcripts sharing each gene ID, and differential expression was tested based on the false discovery rate-adjusted *P*-value at the 5% significance level using Cuffdiff (Trapnell et al. 2012). Gene names and annotations (gene ontology biological processes) of DEGs were examined using Metascape (http://metascape.org/gp/index.html#/main/step1).

### RNAi-mediated Knockdown

Total RNA was extracted from a *C. b. fortunei* final instar larva using the RNeasy Mini Kit and reverse-transcribed to cDNA using the ReverTra Ace -α- Kit (Toyobo, Osaka, Japan). PCR was performed using cDNA and the following primer set to amplify a 502-bp portion of *C. b. fortunei opa* exon 1 and 2 sequences:

5′-TAATACGACTCACTATAGGGAGATGAACATGTCGGTGGACCTG-3′ and5′-TAATACGACTCACTATAGGGAGAAGGACTTGTCGCACCGTTAG-3′. These primers contained the T7 RNA polymerase promoter sequence at their 5′ ends. The PCR product was concentrated by ethanol precipitation and purified using the QIAquick Gel Extraction Kit (Qiagen). dsRNA was synthesized from the PCR product using the MEGAscript T7 Kit (Invitrogen, Carlsbad, CA, USA) and dissolved in RNase-free water. A dsRNA fragment of the *GFP* gene sequence was synthesized and used as a control.

The dsRNA solution was injected into *C. b. fortunei* final instar larvae before the prepupal stage using a FemtoJet 4i (Eppendorf, Hamburg, Germany) with a Narishige glass capillary on a micromanipulator (Narishige, Tokyo, Japan). A total of 5 μL dsRNA (0.1 to 2.0 μg/μL) was injected into the abdominal parts (supplementary table S4, Supplementary Material online). The injected larvae were reared until approximately the 30-day-old adult stage; rearing conditions were as previously described (Konuma et al. 2013b, 2014). The head and thoracic dimensions were measured using digital calipers. The geometric mean of all the measurements was used as a metric for body size (Mosimann 1970; Jungers et al. 1995; Konuma et al. 2013b, 2014). Size correction of the measurements was performed using Burnaby's procedure (Burnaby 1966; Klingenberg 1996; Konuma et al. 2013b, 2014). Burnaby's procedure is a general method for removing body size effects from multivariate morphometric measurements consisting of multiple groups (Klingenberg 2016). Burnaby's procedure project data points in a space perpendicular to the body size axis. By applying Burnaby's back-projection method (McCoy et al. 2006) to log-transformed measurements, we were able to obtain morphometric values adjusted so that the log-transformed body size is zero [i.e. body size = one (Klingenberg 1996). The morphological differences between *opa*-RNAi-treated individuals and controls were tested at the 5% significance level using ANOVA.

### Phylogenetic Analysis of Opa Orthologues

We conducted a phylogenetic analysis for the amino-acid sequences of Opa orthologues from 12 animals: *Mus musculus* Zic1 (Aruga et al. 1996), NP_033599.2; *Octopus ocellatus* Zic, BAE94136.1; *Parasteatoda tepidariorum* Opa (Kanayama et al. 2011), BAK93299.1; *Papilio xuthus* Opa, XP_013167317.1; *Bombyx mori* Opa, XP_004924712.2; *Drosophila melanogaster* Opa (Benedyk et al. 1994), NP_524228.2; *Anopheles gambiae* Opa, XP_321856.5; *Tribolium castaneum* Opa (Choe et al. 2017), AQY46990.1; *Carabus uenoi* Opa (Fujisawa et al. 2019); *Carabus japonicus* Opa (Komurai et al. 2017); *Carabus blaptoides fortunei* Opa; and *Carabus blaptoides* capito Opa. Using MEGA ver. 10.1.7 (Kumar et al. 2018), these orthologue sequences were multiple-aligned using CLUSTALW, and a maximum-likelihood analysis was performed on the 224 positions with no gaps (of 751 aligned positions) using the Dayhoff + gamma substitution mode selected based on the Bayesian information criterion; node support values were obtained using a bootstrap analysis with 1,000 replications.

### Resequencing and Comparison of *Opa* Sequences

We conducted PE sequencing using the Illumina HiSeq 1500 on six wild individuals each of *C. b. fortunei* and *C. b. capito* (supplementary table S8, Supplementary Material online). Additional PE sequencing was conducted using HiSeq X Ten on 16 wild individuals of each subspecies. The sequenced reads were preprocessed using Trimmomatic v0.32, and variant calling in the *opa* and 5-kb flanking sequences was performed according to the New York University variant calling pipeline (https://gencore.bio.nyu.edu/variant-calling-pipeline/). To compare the protein structure of Opa between *C. b. fortunei* and *C. b. capito*, we examined the amino-acid sequences of the open reading frame of *Cbf*-Opa and *Cbc*-Opa. To search for CNSs in the *opa* gene region of *Carabus* beetles, we compared *opa* and the 5-kb flanking sequences among *C. b. fortunei*, *C. b. capito*, *C. japonicus* (Komurai et al. 2017), and *C. uenoi* (Fujisawa et al. 2019) using mVISTA (http://genome.lbl.gov/vista/index.shtml). A short region was predicted as a gene 171 bases upstream of *opa* in *C. b. fortunei*, but no corresponding region was found in *C. japonicus* and *C. uenoi*. Since the gene prediction of this region was supported only by GeneMark and not by Cufflinks or Augustus, it was considered a possible false positive (Lukashin and Borodovsky 1998); therefore, we did not assume that this region represented a coding sequence. The Gumby algorithm (Prabhakar et al. 2006) of mVISTA was used to estimate neutral evolutionary rates from non-coding sequences in multiple sequence alignments and then identify conserved (slowly evolving) local segments using LOD scores of constrained evolution. The LOD scores were phylogenetically weighted as conservation scores and translated into *P*-values using Karlin–Altschul statistics (Karlin and Altschul 1990): conservation score = −log_10_ (*P*-value). We defined sequence regions with a conservation score greater than –log_10_ 0.05 as CNSs.

### Selective Sweep

To verify the hypothesis that a selective sweep (Smith and Haigh 1974) occurred at the location of the *opa* sequence of *C. b. capito*, we examined Zeng's *E* value (Zeng et al. 2006), standardized Fay and Wu's *H*-value (Fay and Wu 2000; Zeng et al. 2006), and Tajima's *D* (Tajima 1989) on the sequence of *Cbc*-*opa* using equations 13, 11, and 4 in the study by Zeng et al. (2006), respectively. Zeng's *E* is high and Fay and Wu's *H* is low in the presence of numerous high-frequency alleles derived from ancestors, whereas Tajima's *D* is low upon the occurrence of many low-frequency alleles such as singletons. These features of the allele frequency spectrum indicate the footprint of a selective sweep. First, we identified the location where Zeng's *E*-value was the highest and the standardized Fay and Wu's *H* and Tajima's *D*-values were the lowest in the 2,000-bp sliding windows. A peak shift from a slender to a stout body shape appeared to have occurred after the ancestor of *C. b. capito* colonized Sado Island (Konuma et al. 2013a). Therefore, *Cbf*-*opa* was used as an ancestral sequence in Zeng's *E* and standardized Fay and Wu's *H*. Then, we compared the *E*, *H*, and *D* peak values with their empirical distributions generated using coalescent simulations under a neutral infinite-site model. A total of 10,000 simulations were conducted using DnaSP v6.12.03 (Rozas et al. 2017), in which the theta parameter = 7.6 × 10^−3^ and recombination parameter = 2.5 × 10^−1^. The theta parameter represents genome-wide average nucleotide diversity, and the recombination parameter is theta × recombination rate/2.9 × 10^−9^, based on the study by Liu, Jia et al. (2017). The recombination rate was estimated to be 9.5 × 10^−8^, according to the genetic map length (1.8 × 10^1^)/genome size (1.9 × 10^8^). In the simulations, the population size was assumed to be constant and large. We estimated *P*(Sim ≤ Obs), the probability that the simulated values were equal to or lower than the observed values. The observed values were significant if *P*(Sim ≤ Obs) ≥ 0.95 in Zeng's *E* and if *P*(Sim ≤ Obs) < 0.05 in the standardized Fay and Wu's *H* and Tajima's *D*. We also calculated Zeng's *E*, standardized Fay and Wu's *H*, and Tajima's *D* for the entire QTL region to see if selective sweeps are observed for other DEGs in the QTL.

### Phylogenetic Analysis of *C. blaptoides Opa*

We sequenced part of the CNS10 region (total aligned positions: 1,299 bp, comprising the last 415 bp of the CDS and 884 bp of 3′-UTR sequence) for seven subspecies of *C. blaptoides*, including the two focal subspecies, and performed a maximum-likelihood phylogenetic analysis using IQ-TREE version 2.03 with the MFP + MERGE option (i.e. to find the best partitioning scheme followed by tree inference). Node supports were accessed using the Shimodaira–Hasegawa-like approximate log-likelihood ratio test (SH-aLRT) and ultrafast bootstrap analyses, each with 1,000 replications. We used 17 sequences from seven subspecies: *C. b. rugipennis* (*n* = 1), *C. b. viridipennis* (*n* = 3), *C. b. babaianus* (*n* = 2), *C. b. oxuroides* (*n* = 3), *C. b. blaptoides* (*n* = 1), *C. b. capito* (*n* = 3), and *C. b. fortunei* (*n* = 4, including the reference genome sequence). *Carabus* (*Acoptolabrus*) *gehinii* was used as the outgroup.

### Prediction of Transcription Factor Binding Sites

To determine the presence of transcription factor binding sites within the CNS10 region, we searched for transcription factors using JASPAR (https://jaspar.elixir.no/) and examined predicted binding sequences. We used the latest CORE collection data for Insecta (Release 10) from the JASPAR database to identify transcription factors that are predicted to bind to the CNS10 sequence of *C. b. fortunei*. Among the predicted sequences, we focused on those with a relative score of 0.95 or higher based on PWMScan (Ambrosini et al. 2018) and examined the presence/absence of SNPs in those sequences for *C. b. capito*, which were identified using the resequencing data.

## Supplementary Material

msae110_Supplementary_Data

## Data Availability

Genomic sequence data have been deposited in the DNA Read Archive of the DNA Data Bank of Japan (DDBJ; DRA005339, DRA013992, DRA015164, and DRA015293; Extended Data Table 1). The draft genome of *C. b. fortunei* was deposited at DDBJ Annotated/Assembled Sequences (BSRF01000001–BSRF01042502). Sequence data of the partial opa CNS10-CNS11 region were deposited in the DDBJ (accession numbers, LC05474–LC705522). All data are available in the main text or the supplementary materials.
