# Supplementary material for: Odd-Paired is Involved in Morphological Divergence of Snail-Feeding Beetles
Source: Mol Biol Evol. 2024 Jun 10;41(6):msae110. doi: 10.1093/molbev/msae110 (PMC11214159; doi:10.1093/molbev/msae110)
Supplement: msae110_Supplementary_Data [file msae110_supplementary_data.pdf]

Supplementary Materials for

odd-paired is involved in morphological divergence of snail-feeding beetles

Junji Konuma,* Tomochika Fujisawa, Tomoaki Nishiyama, Masahiro Kasahara, Tomoko F. Shibata, Masafumi Nozawa, Shuji Shigenobu, Atsushi Toyoda, Mitsuyasu Hasebe, Teiji Sota*

*Corresponding authors

Email: junji.konuma@sci.toho-u.ac.jp (J.K.), sota.teiji.88u@st.kyoto-u.ac.jp (T.S.)

This PDF file includes:

Figs. S1 to S9

Tables S1 to S8

Supplementary references

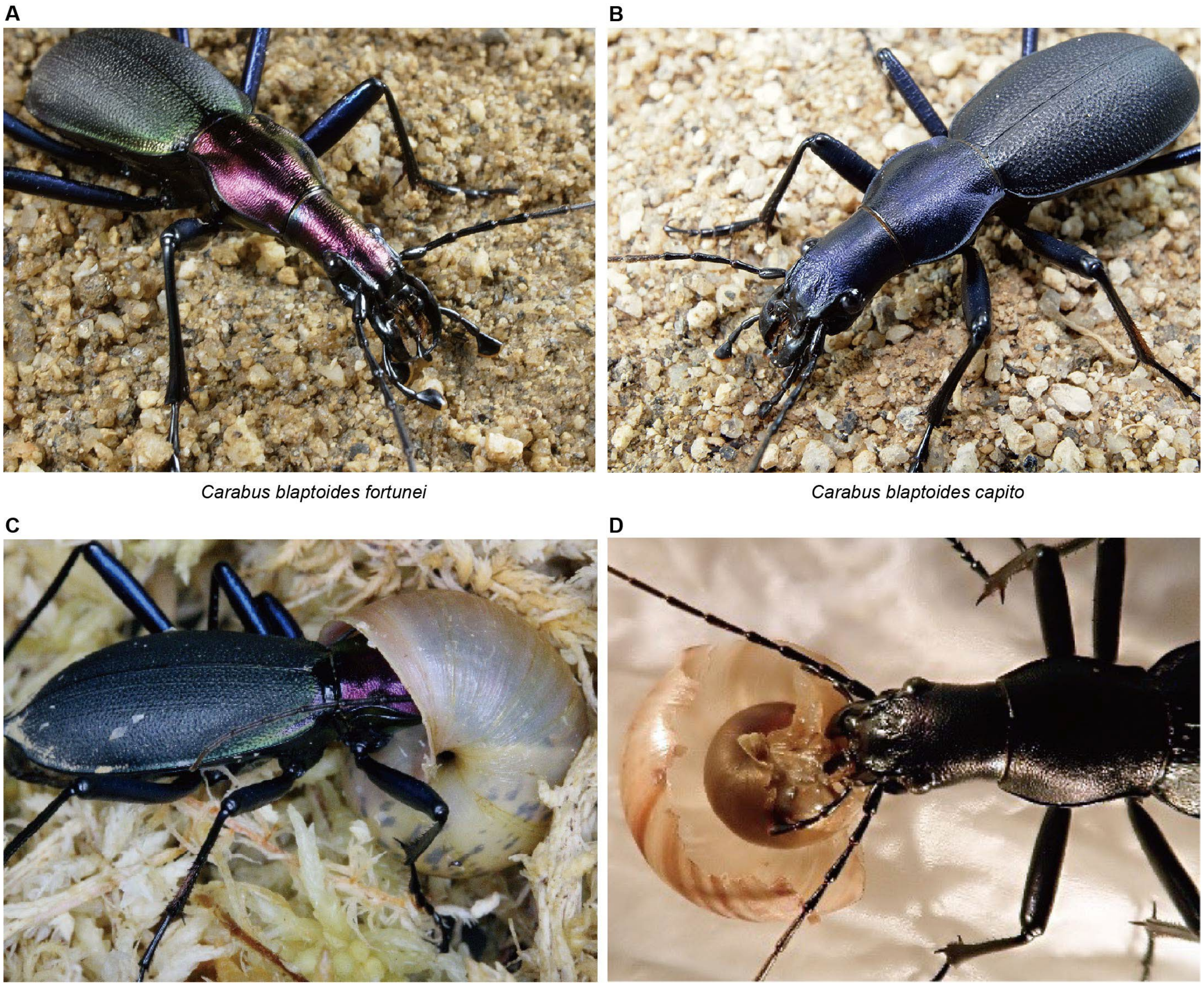

Fig. S1. Slender and stout subspecies of *Carabus blaptoides*. **A**, Slender subspecies (*C. b. fortunei*) from Awashima Island. **B**, Stout subspecies (*C. b. capito*) from Sado Island. **C**, *C. b. fortunei* inserting its head into a shell. Slender beetles can eat large snails using the shell-entry method but cannot eat snails with shell apertures smaller than their heads. **D**, *C. b. capito* crushing the shell through powerful jaw biting. Stout beetles can eat small snails using the shell-crushing method but cannot eat large snails with hard shells. Shell entry and crushing are alternative behaviors for subsisting on snails (Vermeij 1979; DeWitt et al. 2000; Konuma and Chiba 2007; Konuma et al. 2013); this functional trade-off causes diversification in snail-feeding

carabid beetles (Sturani 1962; Ishikawa 1978; Konuma et al. 2011; Konuma et al. 2013; Akiyama et al. 2020; Sota 2022).

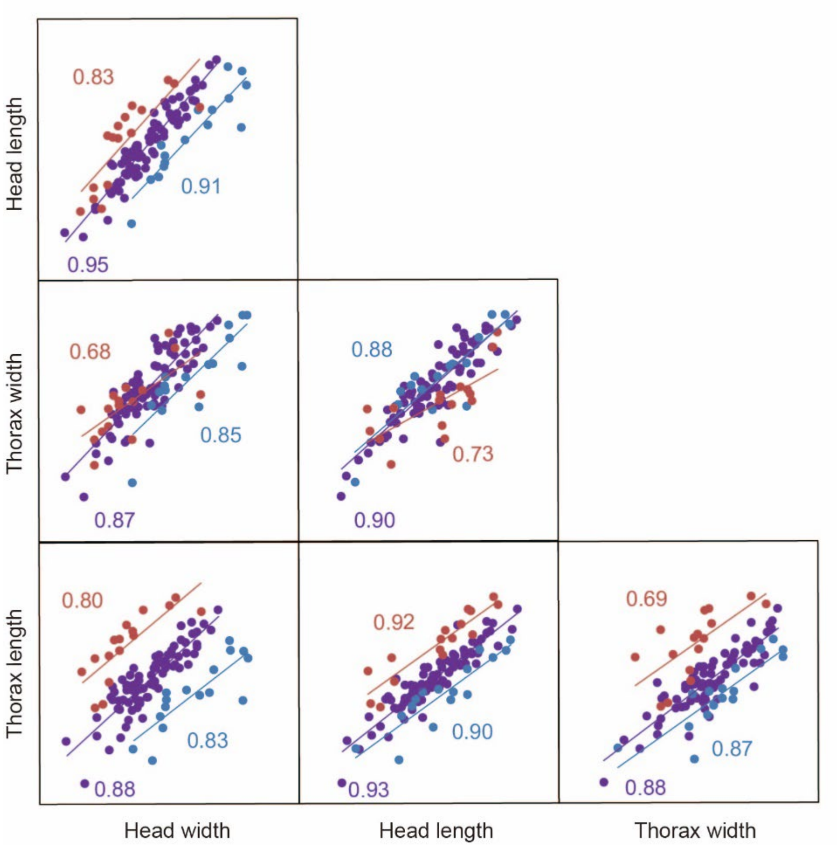

Fig. S2. Scatter plots between the measured dimensions for *C. b. fortunei* (red), *C. b. capito* (blue) and backcrossed individuals of (*C. b. fortunei* × *C. b. capito*) × *C. b. fortunei* (purple). Numerals represent correlation coefficients.

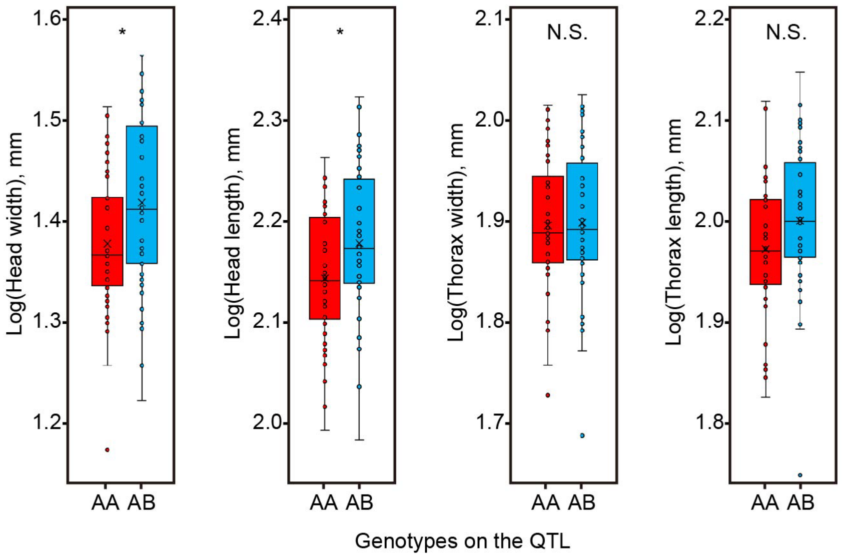

Fig. S3. Morphological differences of the backcrossed individuals between genotypes on the QTL. AA (red, $n = 42$) and AB (blue, $n = 40$) represent homozygous and heterozygous genotypes, respectively, in which A is the slender subspecies (*C. b. fortunei*) allele, while B is a stout subspecies (*C. b. capito*) allele. Box plots show the mean (cross), median (center line), first and third quartiles (box limits), and 1.5 × interquartile range (whiskers). *ANOVA, $F_{1,80} = 5.52$, $P = 0.021$ for head width; $F_{1,80} = 5.27$, $P = 0.024$ for head length. N.S., not significant: ANOVA, $F_{1,80} = 0.029$, $P = 0.86$ for thorax width; $F_{1,80} = 3.27$, $P = 0.074$ for thorax length).

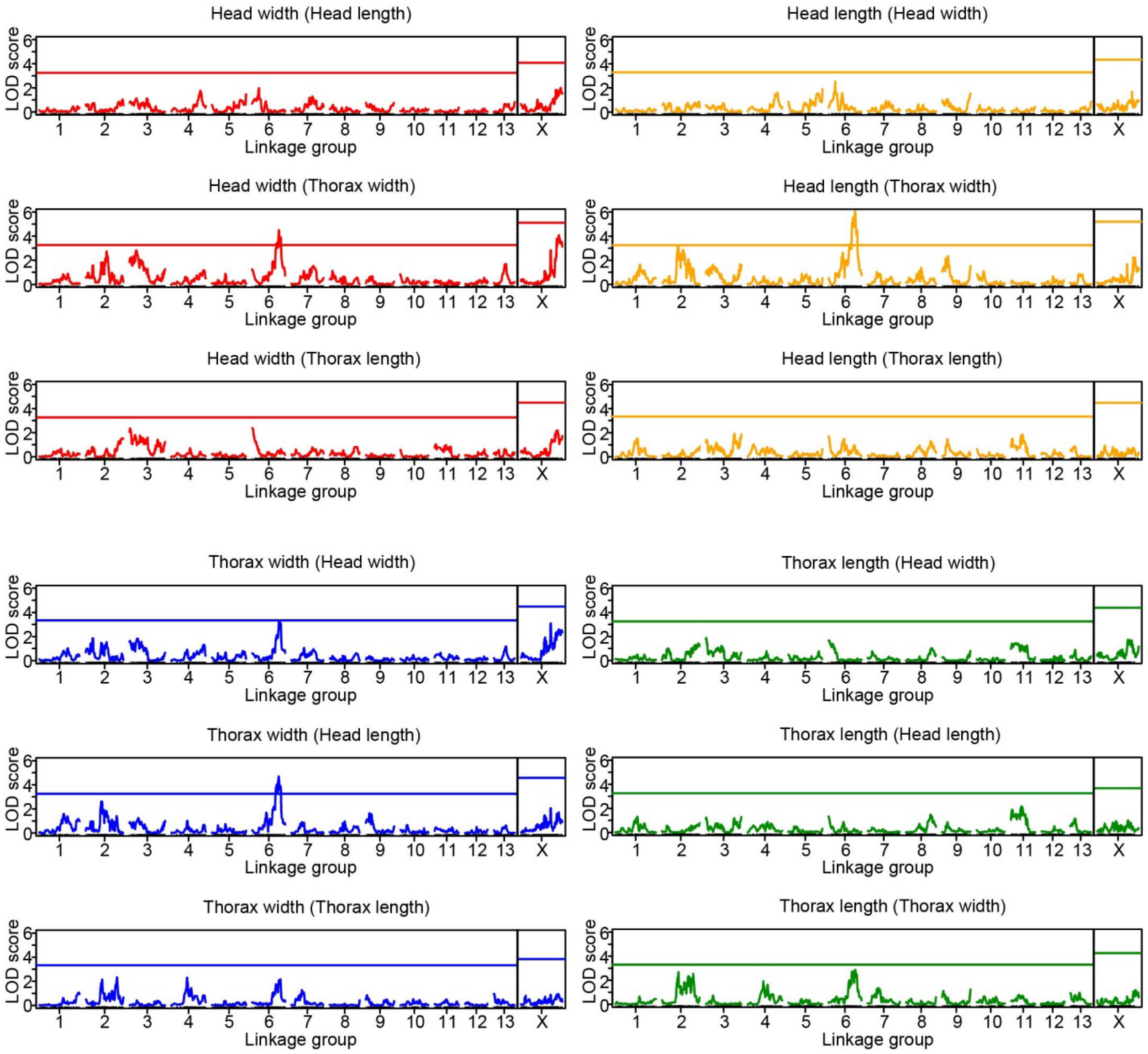

Fig. S4. LOD scores for all morphological dimensions. LOD scores for the head width (red), head length (orange), thorax width (blue), and thorax width (green) calculated using the ‘scanone’ function, with the other dimension (enclosed in parentheses) used as a covariate.

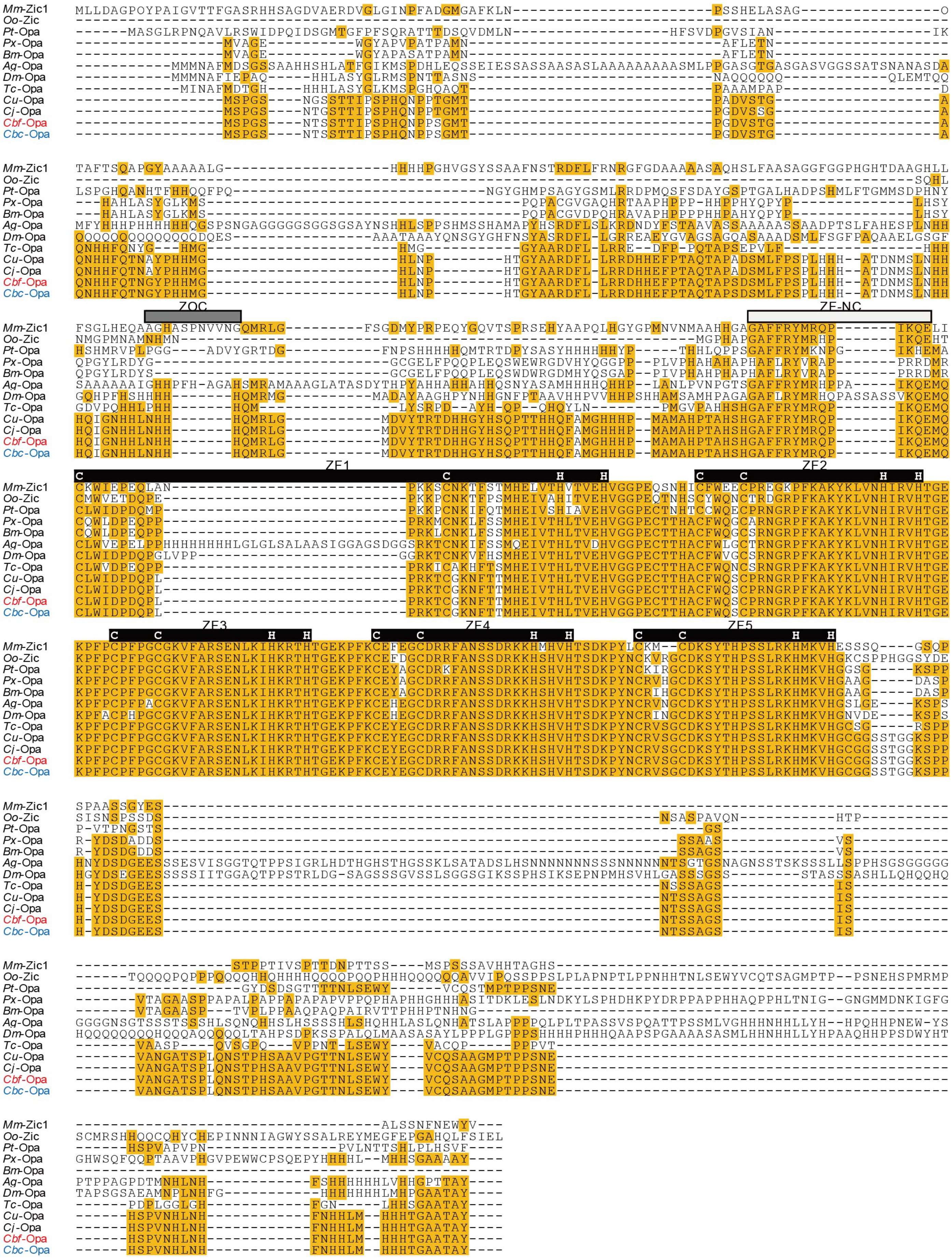

Mm-Zic1
Oo-Zic
Pt-Opa
Px-Opa
Bm-Opa
Ag-Opa
Dm-Opa
Tc-Opa
Cu-Opa
Cj-Opa
Cbf-Opa
Cbc-Opa
ZOC
ZF-NC
ZF1
ZF2
ZF3
ZF4
ZF5

Fig. S5. Aligned Opa amino-acid sequences of the 12 bilaterian species. Letters with orange backgrounds indicate consensus sequences. Five tandem-repeated zinc finger (ZF) domains are shown above the alignments as black bars in which the positions of the C2H2 motifs are indicated with white letters. The ZF domains are structured with two antiparallel β-sheets and an α-helix motif and can bind to a DNA sequence and protein (Wolfe et al. 2000; Aruga et al. 2006). Two other conserved domains, the Zic-Opa conserved (ZOC) and the N-terminal to zinc-fingers conserved (ZF–NC) domains, are indicated using dark and light grey bars, respectively. The ZOC domain is involved in transcriptional activation (Mizugishi et al. 2004), whereas the function of ZF–NC remains unclear (Hursh and Stultz 2018). Taxon names and codes: house mouse, *Mus musculus* (*Mm*-Zic1); octopus, *Octopus ocellatus* (*Oo*-Zic); spider, *Parasteatoda tepidariorum* (*Pt*-Opa); swallowtail butterfly, *Papilio xuthus* (*Px*-Opa); silkworm moth, *Bombyx mori* (*Bm*-Opa); mosquito, *Anopheles gambiae* (*Ag*-Opa); fruit fly, *Drosophila melanogaster* (*Dm*-Opa); red flour beetle, *Tribolium castaneum* (*Tc*-Opa); *Carabus uenoi* (*Cu*-Opa); *C. japonicus* (*Cj*-Opa); *C. b. fortunei* (*Cbf*-Opa; red text); and *C. b. capito* (*Cbc*-Opa; blue text).

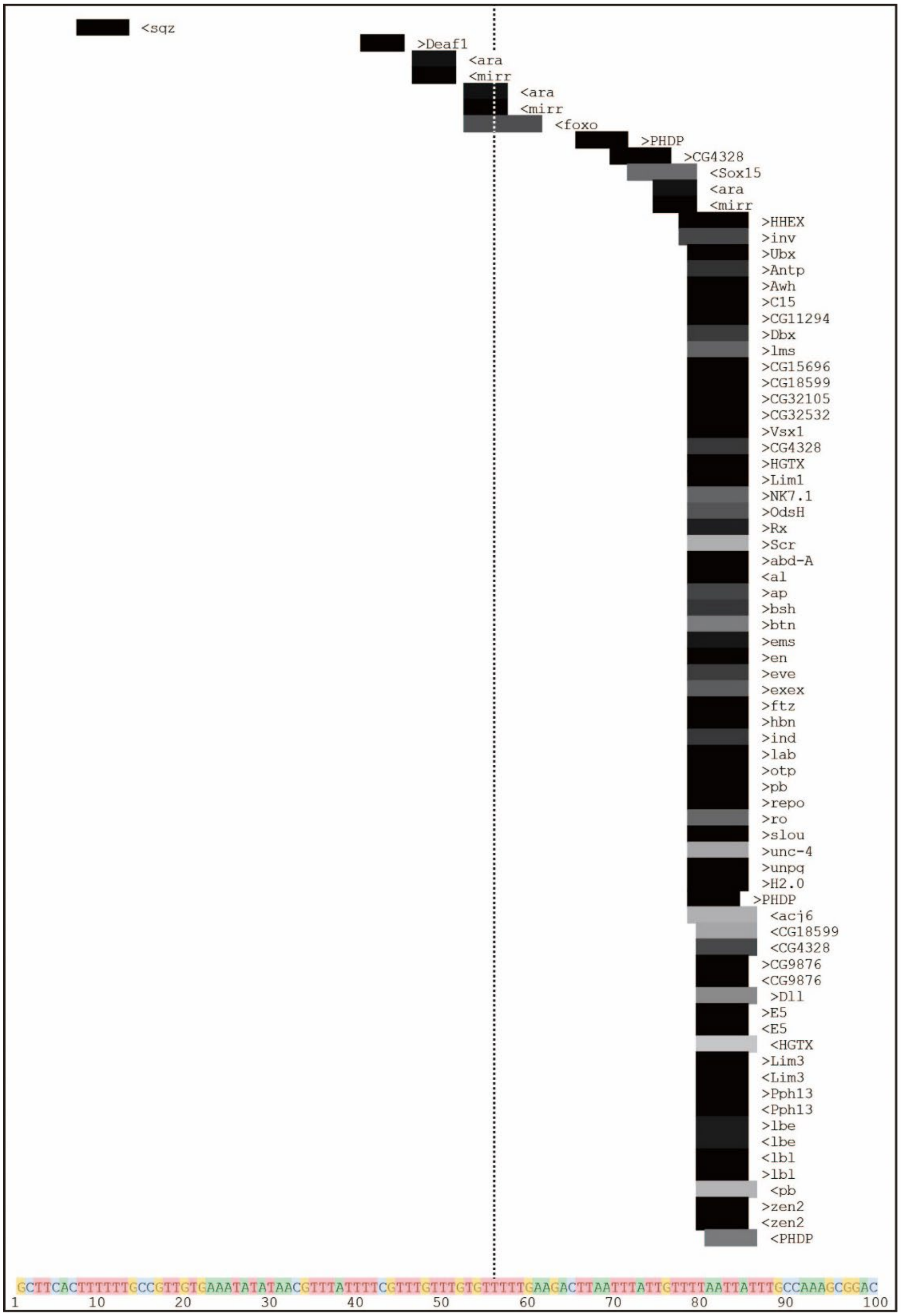

Fig. S6. Predicted transcription factor binding sites within the CNS10 region

The predicted positions of transcription factor binding sites are mapped on the CNS10 sequence.

Binding sites are indicated by horizontal bars, with strand information (>, plus; <, minus) and

transcription factor names. Darker colors of bars represent higher predicted binding scores.

Dashed lines represent positions of SNPs in *C. b. capito*.

Fig. S6 (Continued).

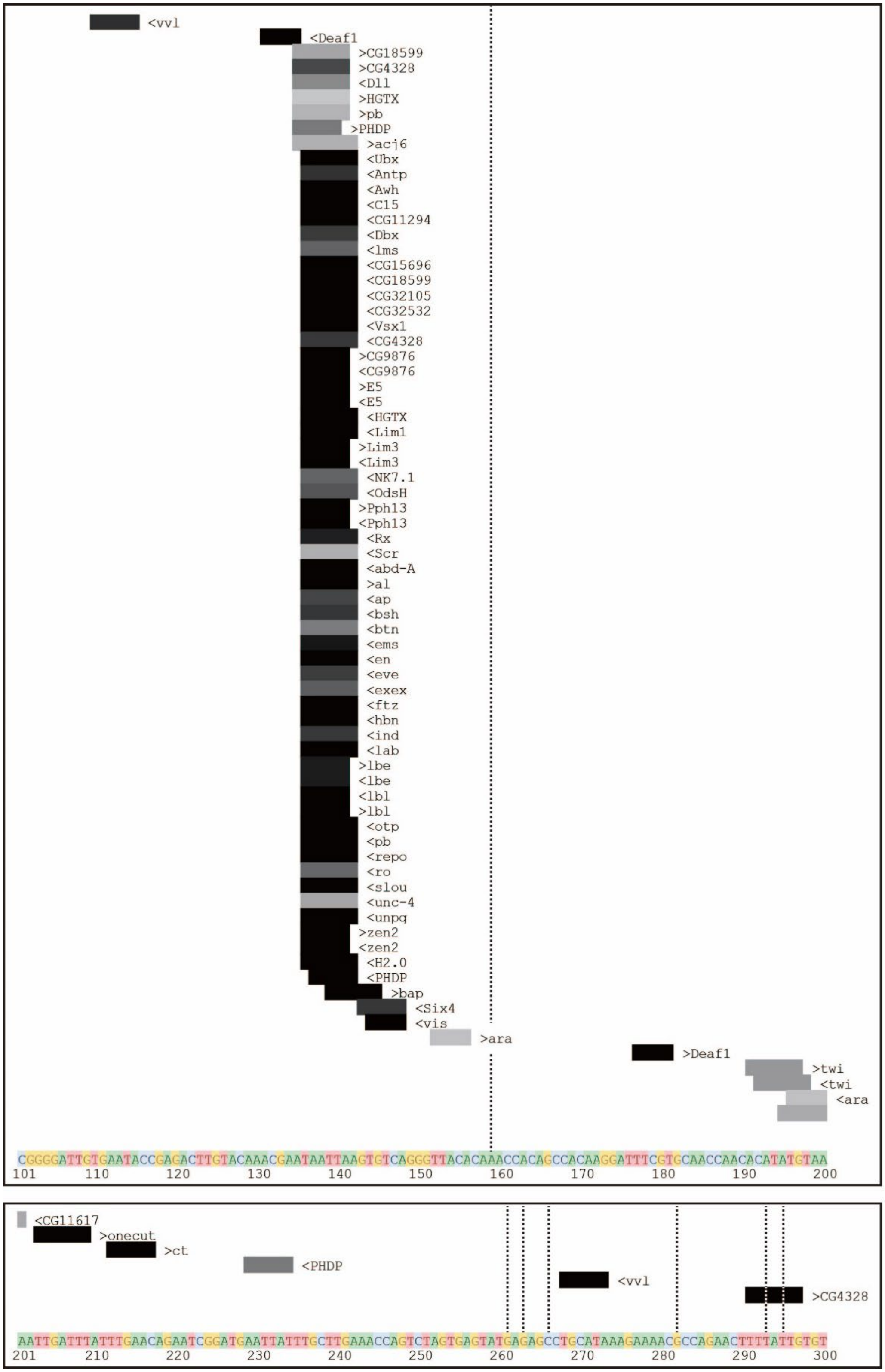

Fig. S6 (Continued).

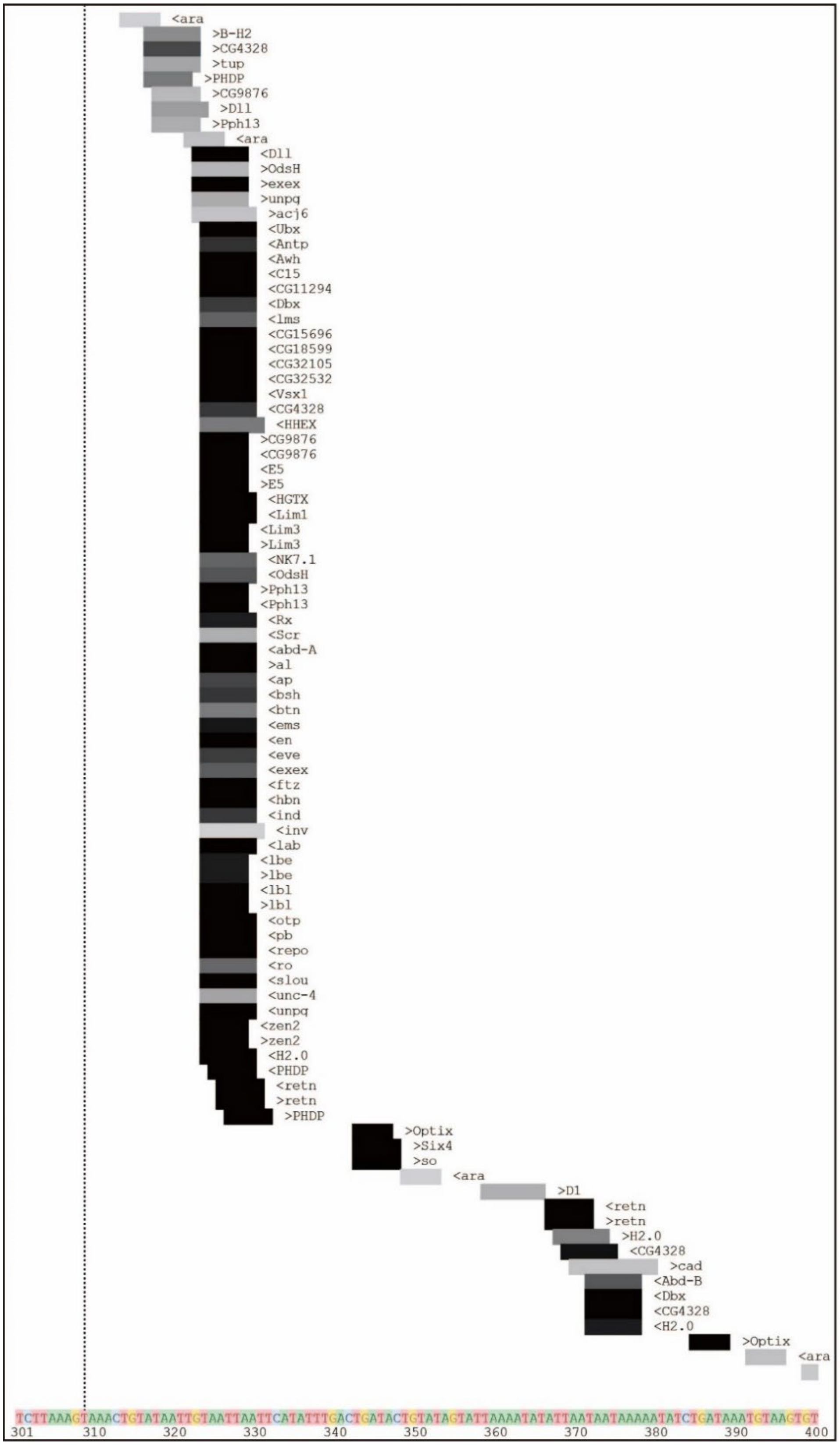

Fig. S6 (Continued).

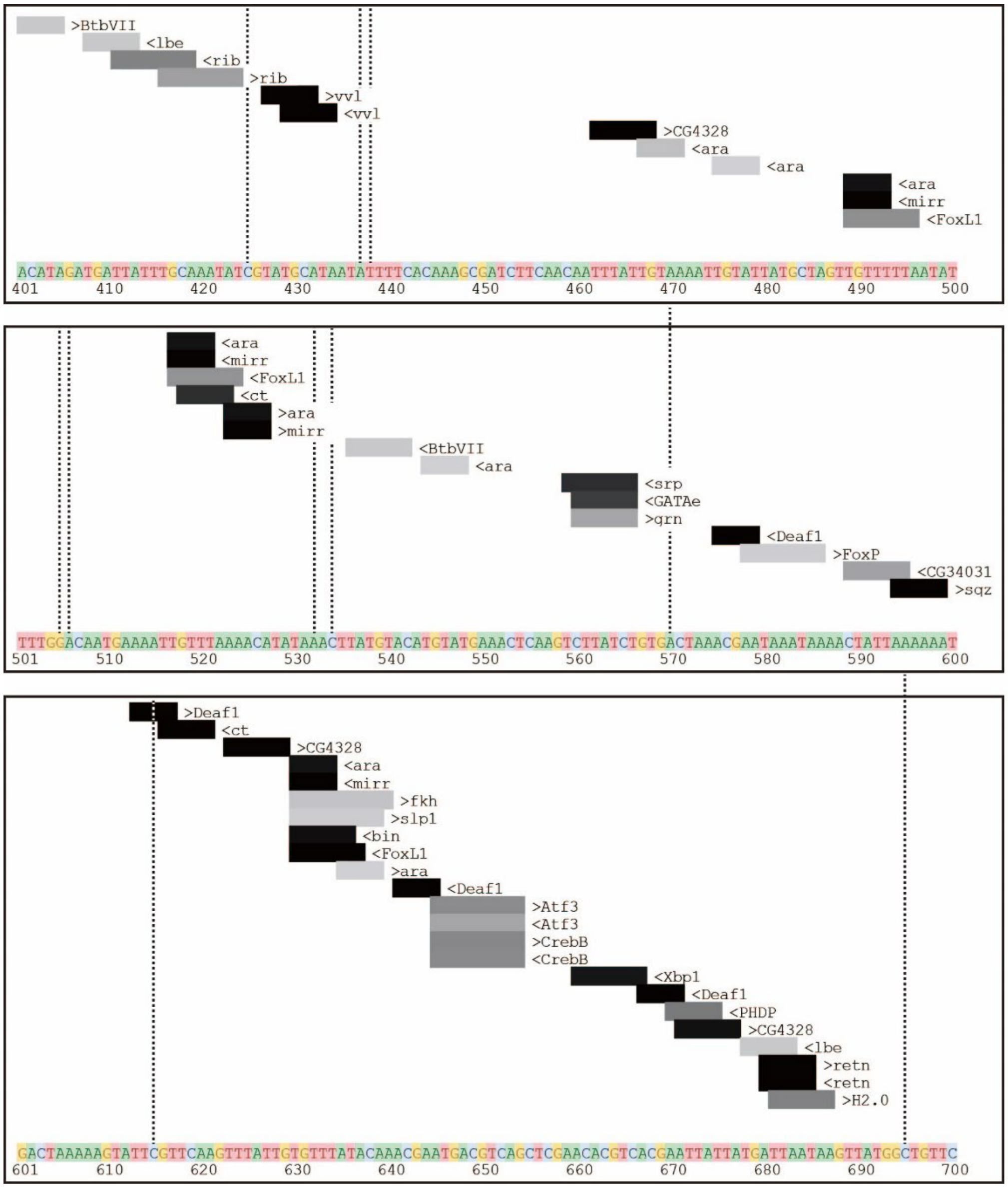

Fig. S6 (Continued).

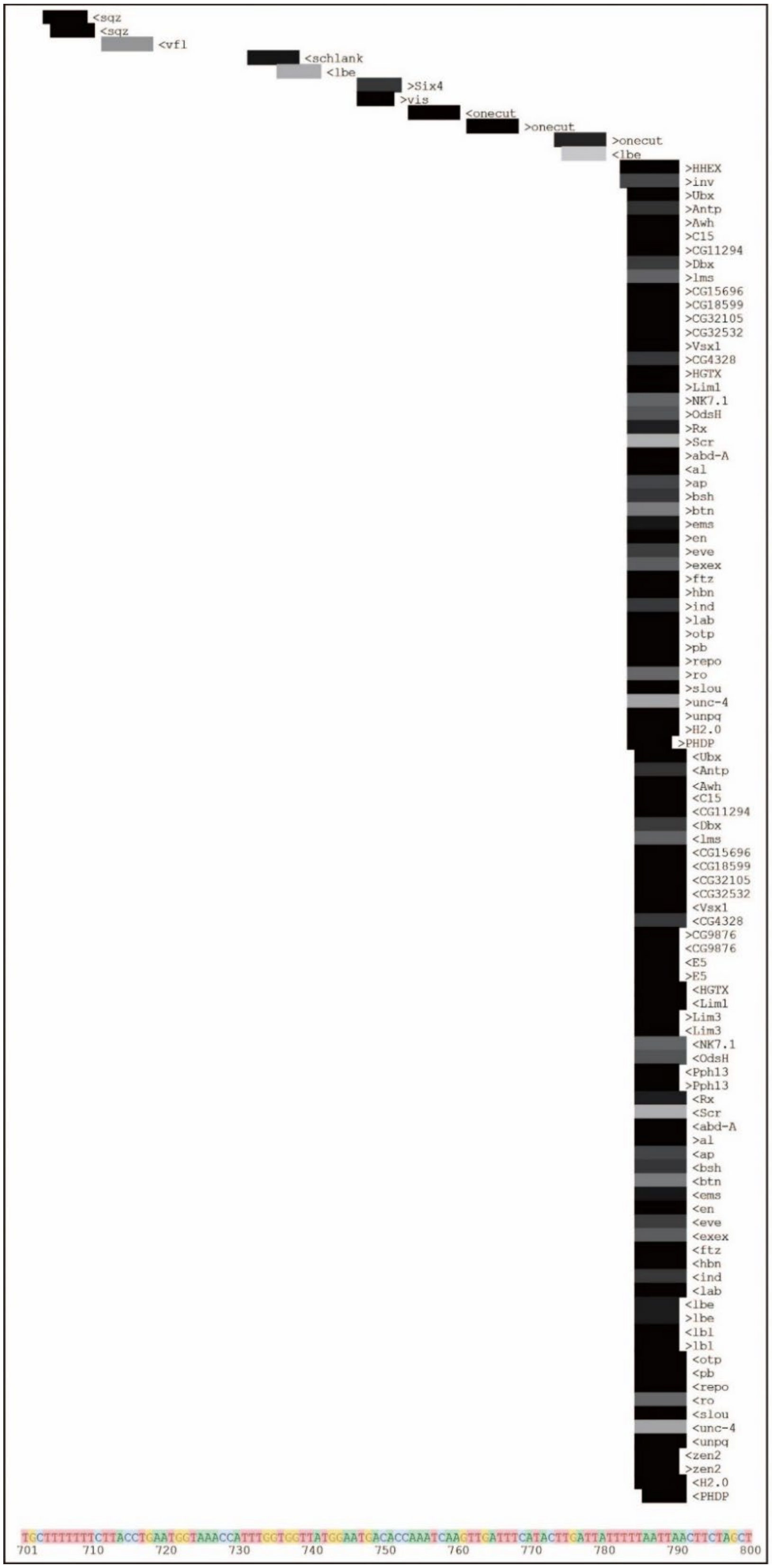

Fig. S6 (Continued).

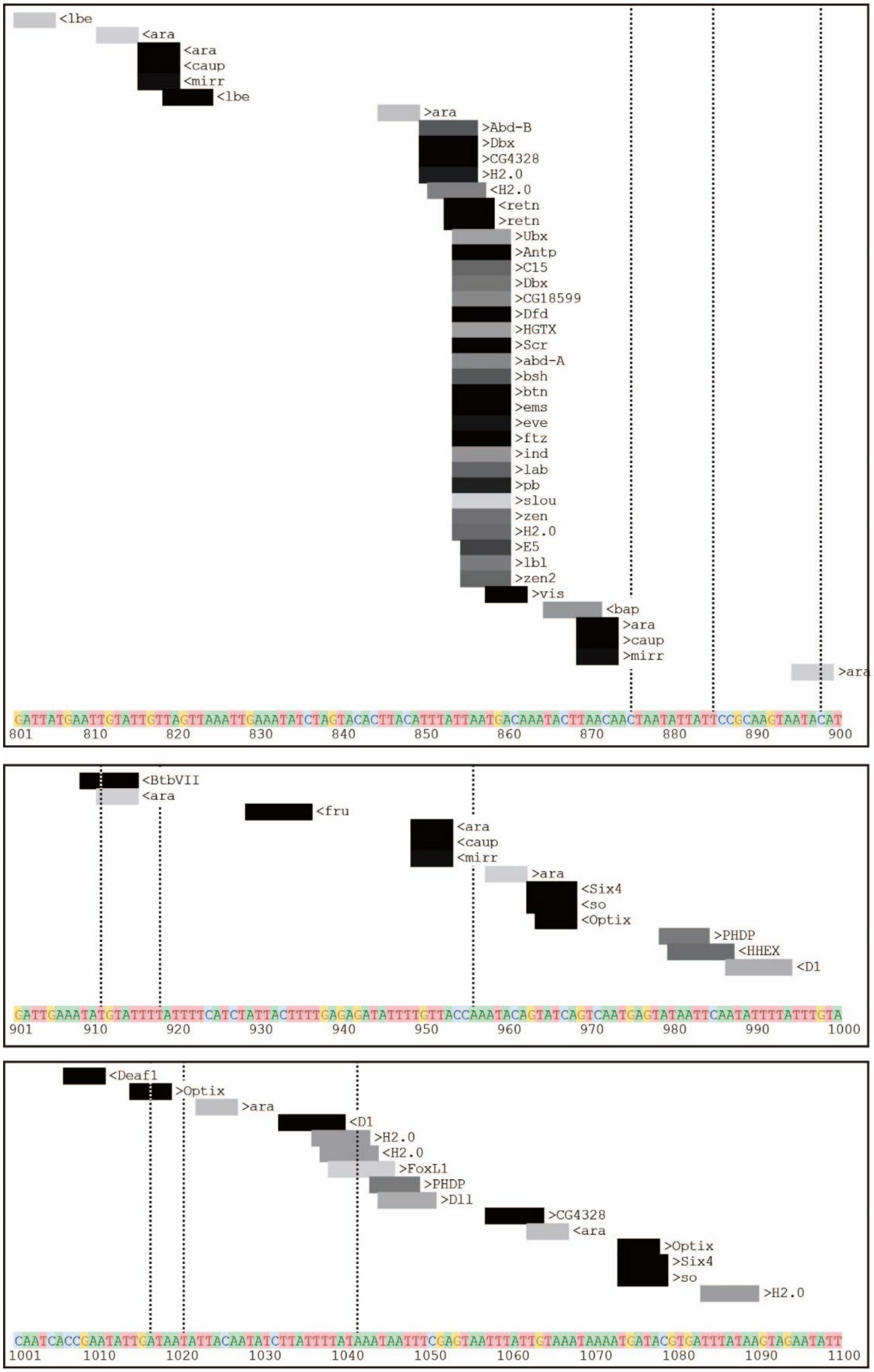

Fig. S6 (Continued).

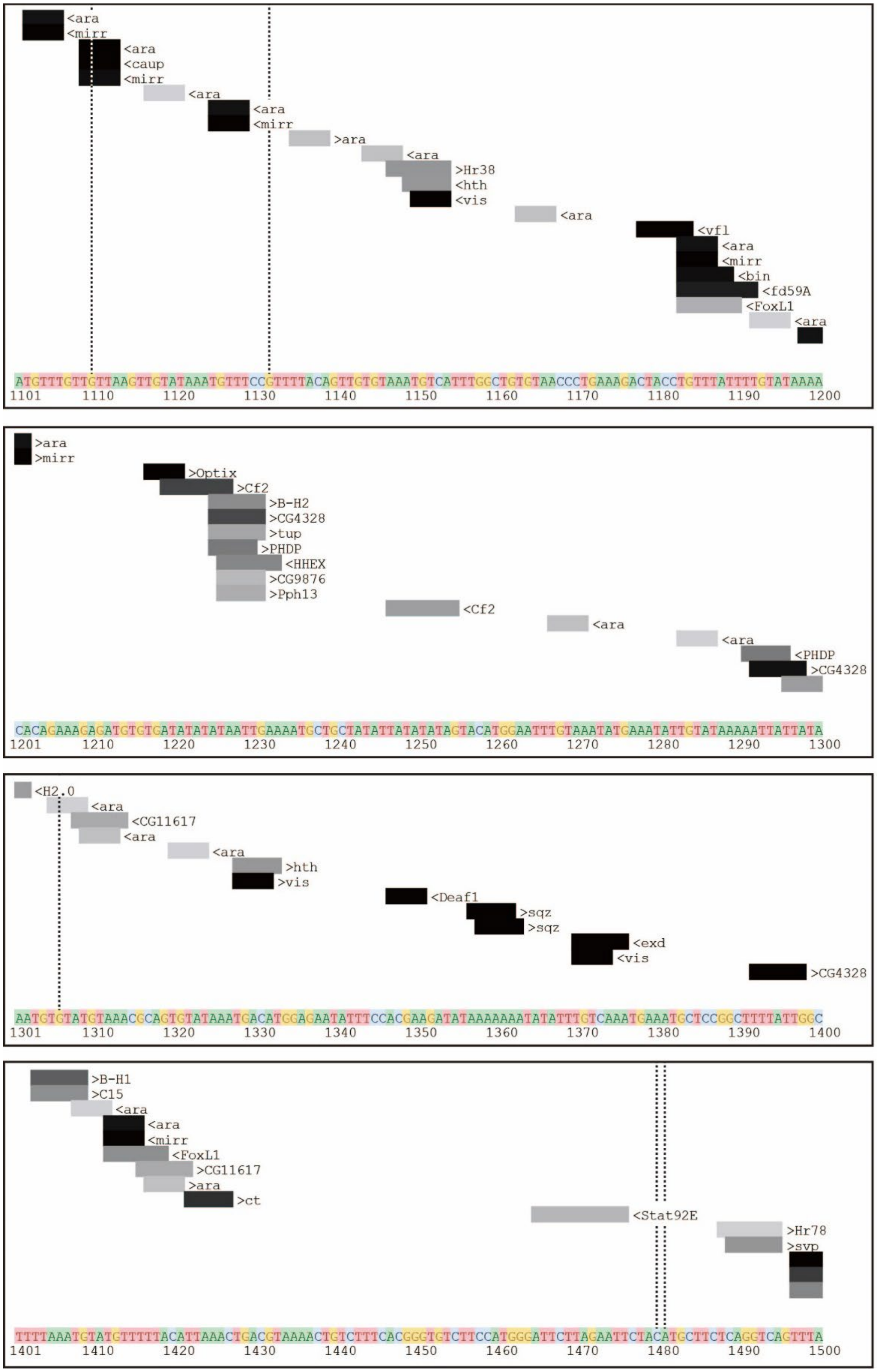

Fig. S6 (Continued).

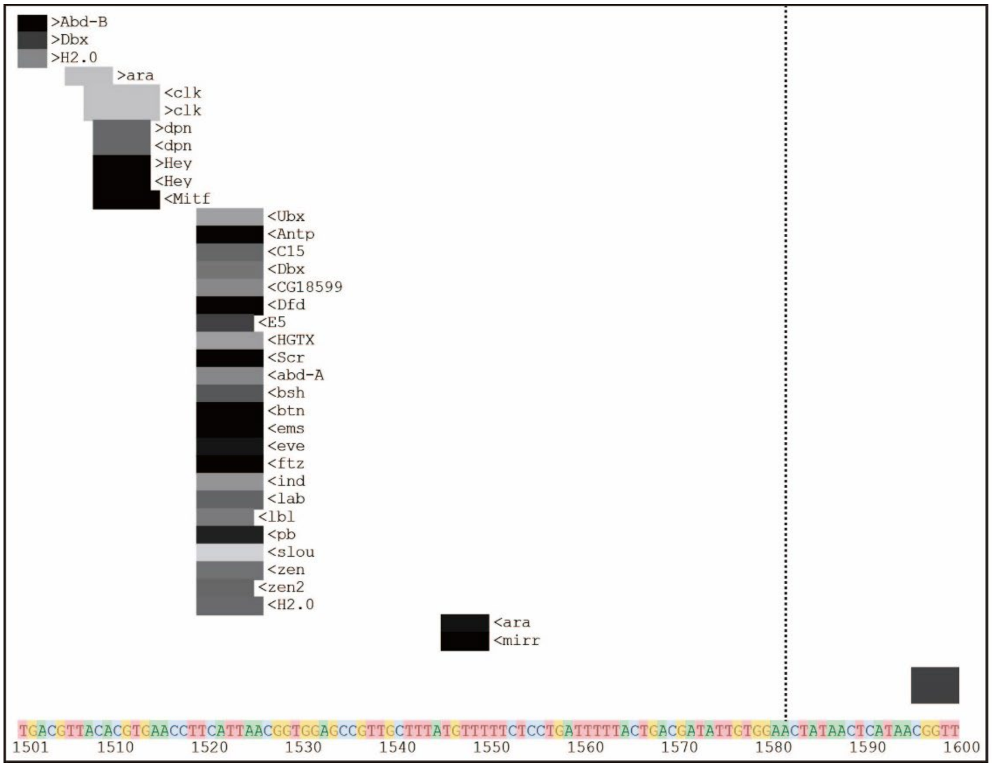

Fig. S6 (Continued).

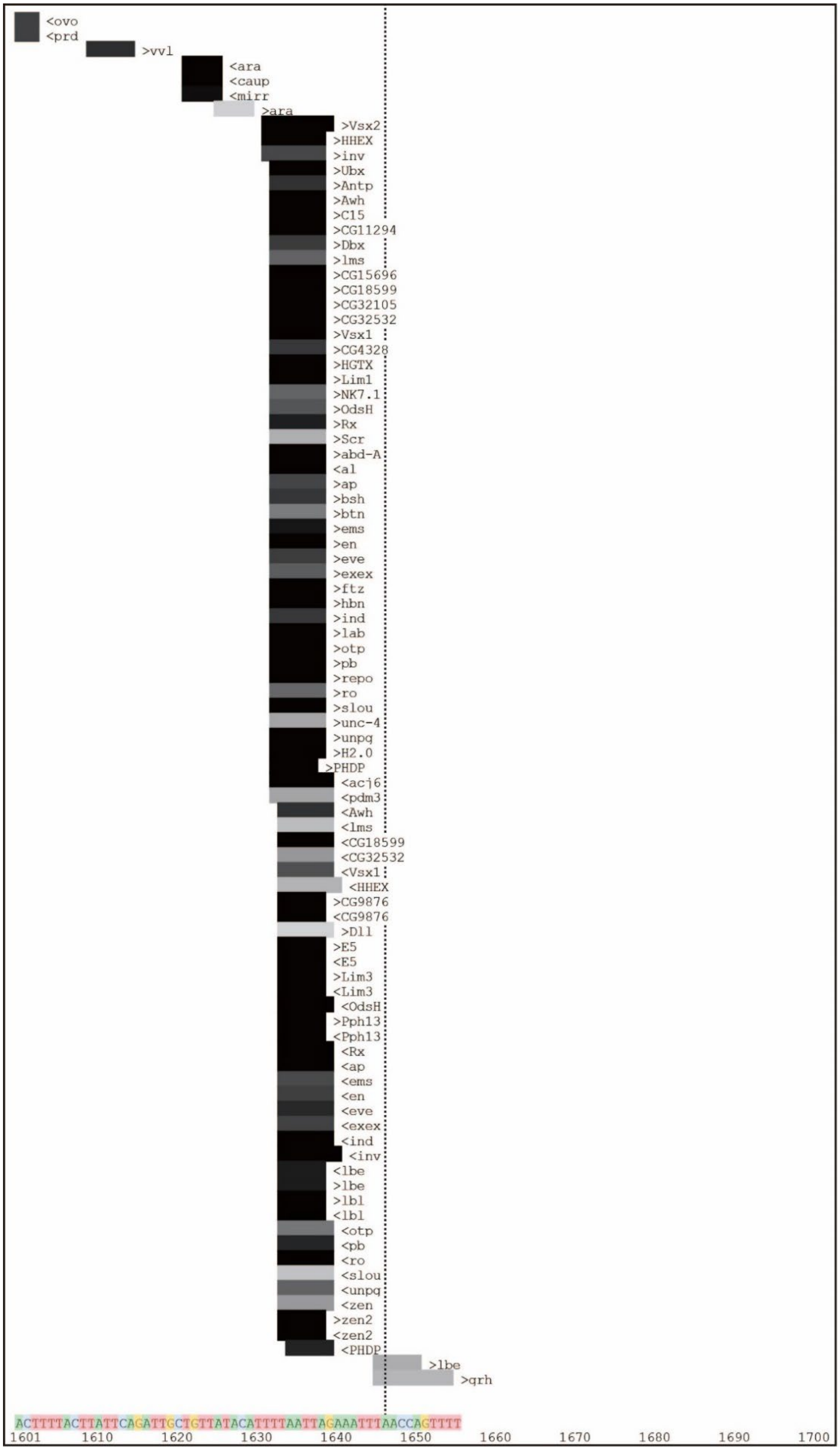

<ovo
<prd
>vvl
<ara
<caup
<mirr
>ara
>Vsx2
>HHEX
>inv
>Ubx
>Antp
>Awh
>C15
>CG11294
>Dbx
>lms
>CG15696
>CG18599
>CG32105
>CG32532
>Vsx1
>CG4328
>HGTX
>Lim1
>NK7.1
>OdsH
>Rx
>Scr
>abd-A
<al
>ap
>bsh
>btn
>ems
>en
>eve
>exex
>ftz
>hbn
>ind
>lab
>otp
>pb
>repo
>ro
>slou
>unc-4
>unpg
>H2.0
>PHDP
<acj6
<pdm3
<Awh
<lms
<CG18599
<CG32532
<Vsx1
<HHEX
>CG9876
<CG9876
>Dll
>E5
<E5
>Lim3
<Lim3
<OdsH
>Pph13
<Pph13
<Rx
<ap
<ems
<en
<eve
<exex
<ind
<inv
<lbe
>lbe
>lbl
<lbl
<otp
<pb
<ro
<slou
<unpg
<zen
>zen2
<zen2
<PHDP
>lbe
>grh
ACTTTTACTTATTCAGATTGCTGTTATACATTTTAATTAGAAATTTAACCAGTTTT
1601
1610
1620
1630
1640
1650
1660
1670
1680
1690
1700

Fig. S6 (Continued).

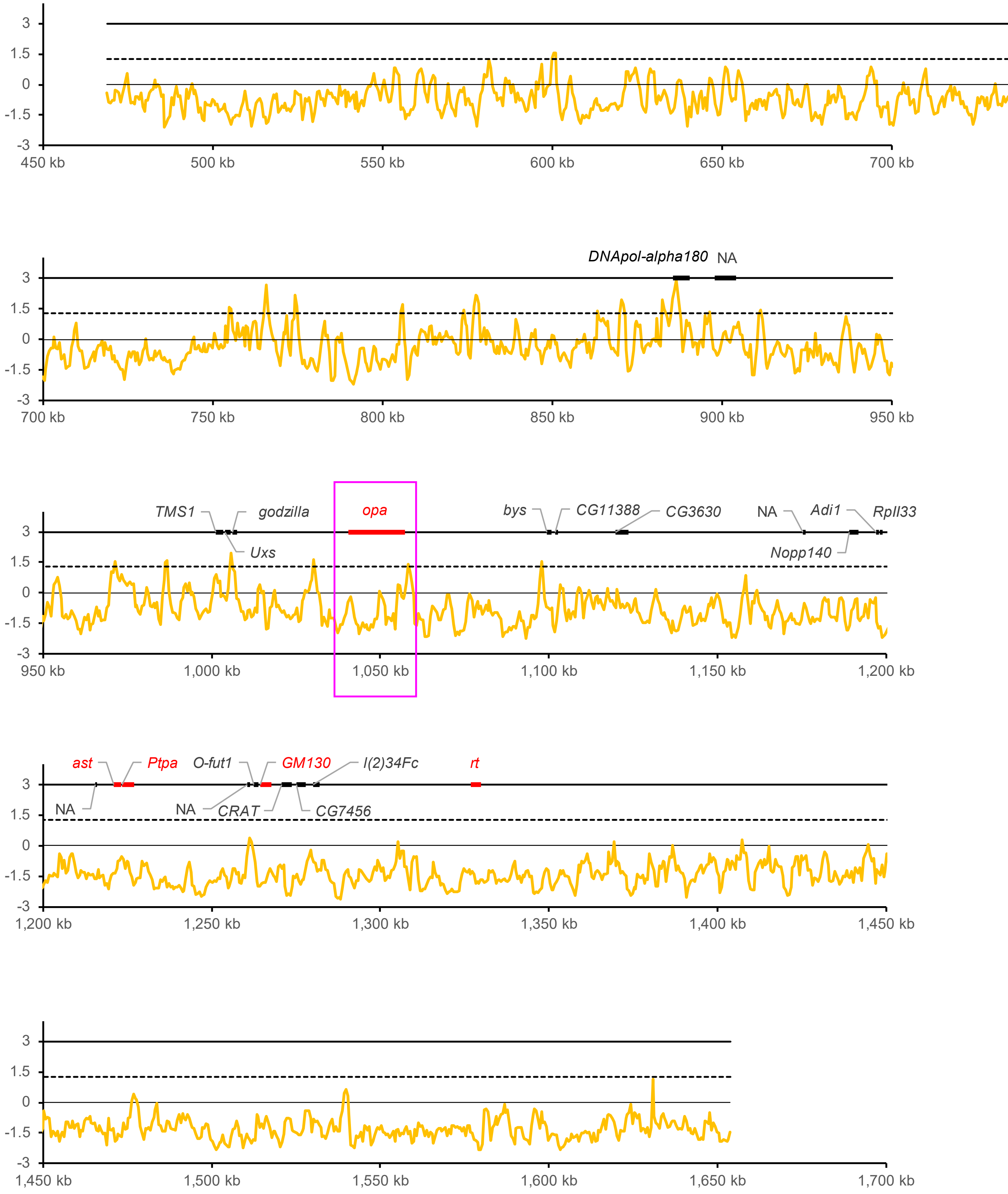
3
1.5
0
-1.5
-3
450 kb
500 kb
550 kb
600 kb
650 kb
700 kb
DNApol-alpha180
NA
700 kb
750 kb
800 kb
850 kb
900 kb
950 kb
TMS1
godzilla
Uxs
opa
bys
CG11388
CG3630
NA
Adi1
RpII33
Nopp140
950 kb
1,000 kb
1,050 kb
1,100 kb
1,150 kb
1,200 kb
ast
Ptpa
O-fut1
GM130
l(2)34Fc
rt
NA
NA
CRAT
CG7456
1,200 kb
1,250 kb
1,300 kb
1,350 kb
1,400 kb
1,450 kb
1,450 kb
1,500 kb
1,550 kb
1,600 kb
1,650 kb
1,700 kb

Fig. S7. Zeng's *E* in quantitative trait loci (QTL).

Yellow plots represent Zeng's *E* obtained by a 2,000-bp sliding window. The magenta rectangle indicates the range examined for *opa* (Fig. 4C). The dashed line represents the 5% significance threshold obtained from the coalescent simulation in the analysis for *opa*. Differentially expressed genes (DEGs) are represented at the top, with red representing DEGs whose gene ontology category is 'developmental process'; NA indicates DEGs that were not found in the RefSeq protein database. The horizontal axis represents the position in scaffold 2061.2.

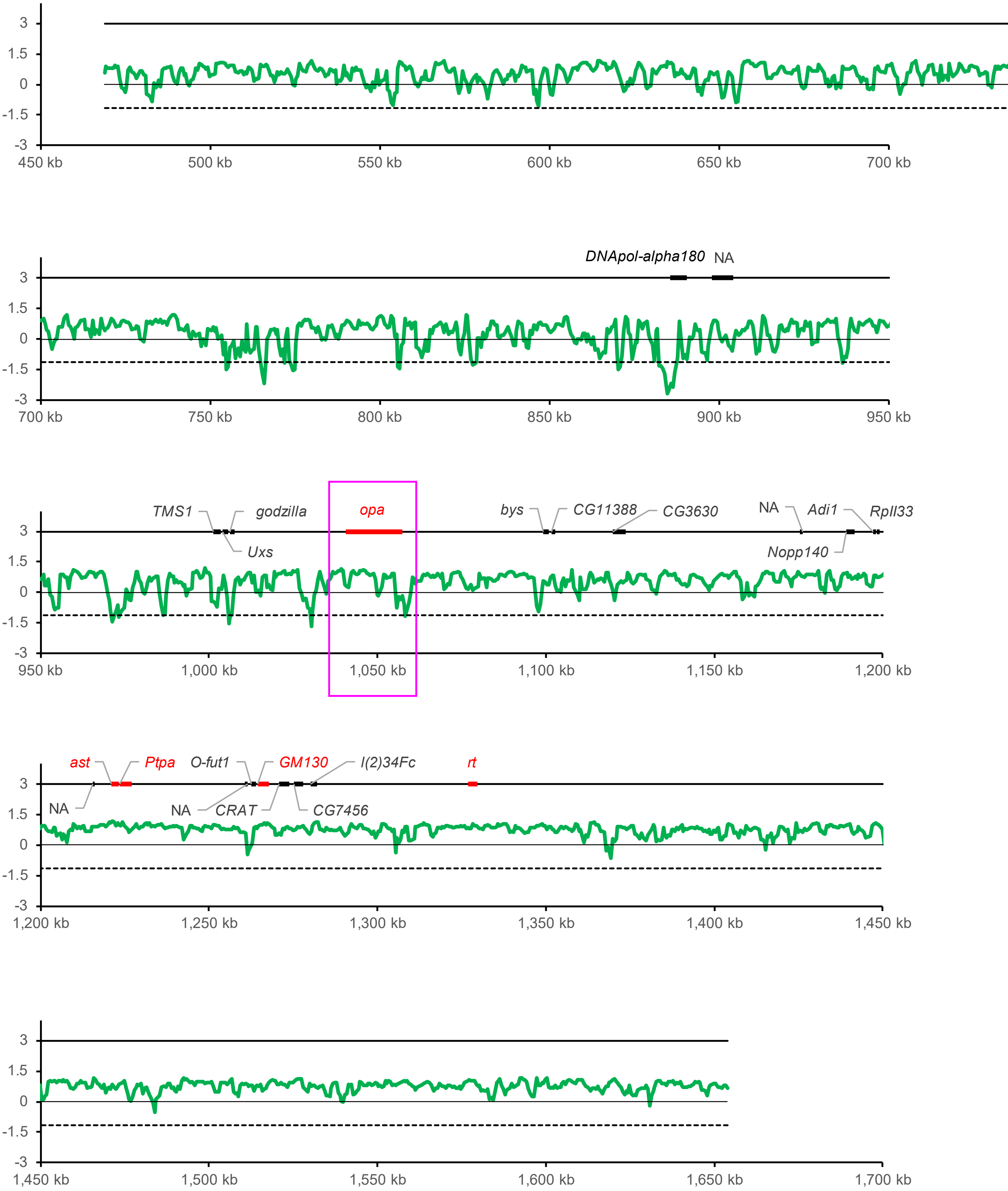
3
1.5
0
-1.5
-3
450 kb
500 kb
550 kb
600 kb
650 kb
700 kb
DNApol-alpha180
NA
700 kb
750 kb
800 kb
850 kb
900 kb
950 kb
TMS1
godzilla
opa
bys
CG11388
CG3630
NA
Adi1
RpII33
Uxs
Nopp140
950 kb
1,000 kb
1,050 kb
1,100 kb
1,150 kb
1,200 kb
ast
Ptpa
O-fut1
GM130
l(2)34Fc
rt
NA
NA
CRAT
CG7456
1,200 kb
1,250 kb
1,300 kb
1,350 kb
1,400 kb
1,450 kb
1,450 kb
1,500 kb
1,550 kb
1,600 kb
1,650 kb
1,700 kb

Fig. S8. Standardized Fay and Wu's *H* in quantitative trait loci (QTL).

Green plots represent standardized Fay and Wu's *H* obtained by a 2,000-bp sliding window. The magenta rectangle indicates the range examined for *opa* (Fig. 4C). The dashed line represents the 5% significance threshold obtained from the coalescent simulation in the analysis for *opa*. Differentially expressed genes (DEGs) are represented at the top, with red representing DEGs whose gene ontology category is 'developmental process'; NA indicates DEGs that were not found in the RefSeq protein database. The horizontal axis represents the position in scaffold 2061.2.

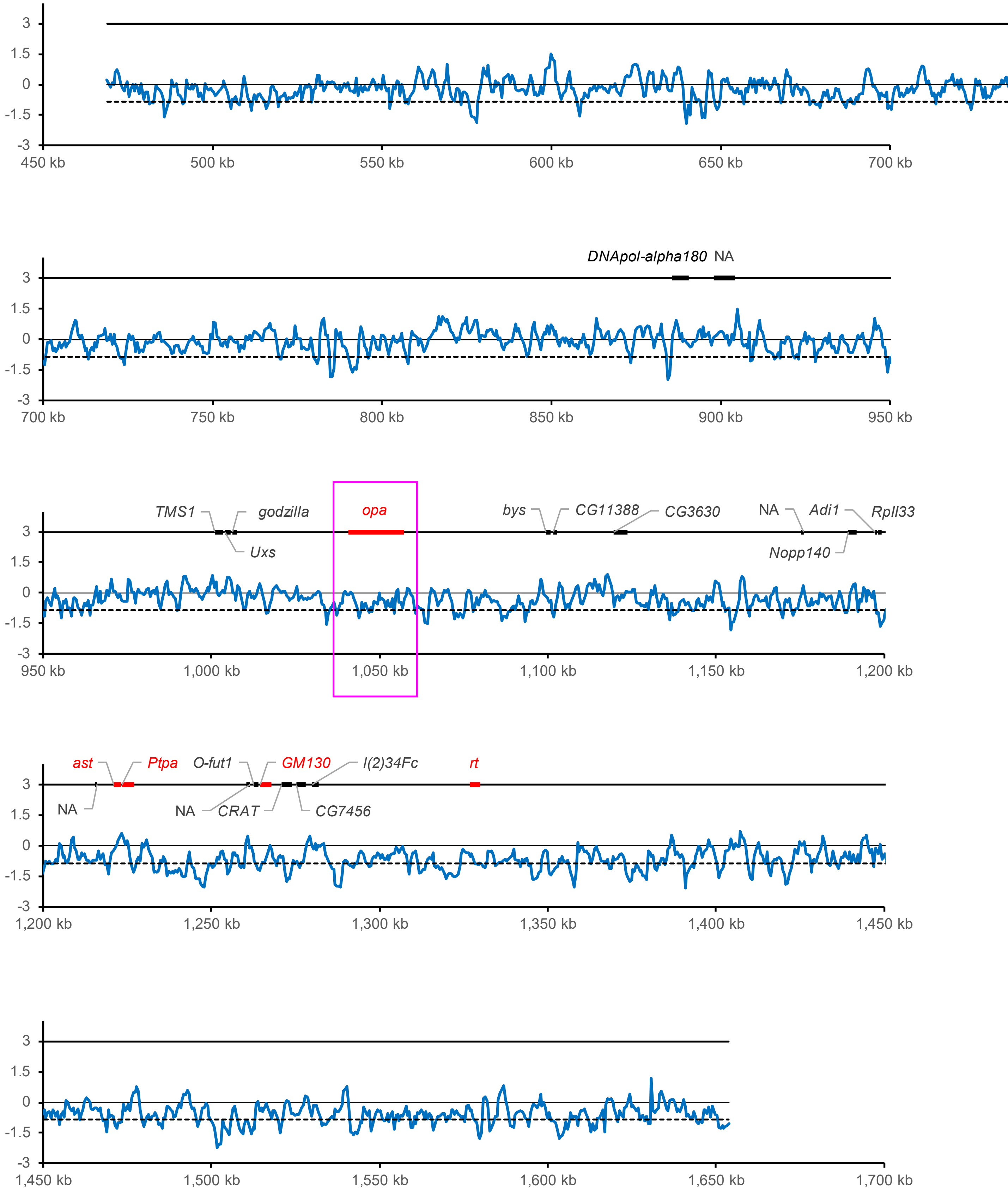

3
1.5
0
-1.5
-3
450 kb
500 kb
550 kb
600 kb
650 kb
700 kb
DNApol-alpha180
NA
700 kb
750 kb
800 kb
850 kb
900 kb
950 kb
TMS1
godzilla
opa
bys
CG11388
CG3630
NA
Adi1
RpII33
Uxs
Nopp140
1,000 kb
1,050 kb
1,100 kb
1,150 kb
1,200 kb
ast
Ptpa
O-fut1
GM130
l(2)34Fc
rt
NA
NA
CRAT
CG7456
1,250 kb
1,300 kb
1,350 kb
1,400 kb
1,450 kb
1,500 kb
1,550 kb
1,600 kb
1,650 kb
1,700 kb

Fig. S9. Tajima's *D* in quantitative trait loci (QTL).

Blue plots represent Tajima's *D* obtained by a 2,000-bp sliding window. The magenta rectangle indicates the range examined for *opa* (Fig. 4C). The dashed line represents the 5% significance threshold obtained from the coalescent simulation in the analysis for *opa*. Differentially expressed genes (DEGs) are represented at the top, with red representing DEGs whose gene ontology category is 'developmental process'; NA indicates DEGs that were not found in the RefSeq protein database. The horizontal axis represents the position in scaffold 2061.2.

Table S1. Sequence data obtained in this study. Read types are denoted as follows: PE, Paired-End; MP, Mate-Pair; SE, Single-End. Coverage was calculated as bases/total scaffold length for DNA-Seq and Re-Seq; called bases/sample size/total exon length for RNA-Seq; number of reads/sample size/number of catalog loci for RAD-Seq.

Sequencing technology	Application	Platform	Read type	Insert size (bp)	No. of reads (M)	Length (bp)	Called base (Gbp)	Sample size	Coverage	Accession No.
DNA-Seq	Assembly	Illumina HiSeq 2000	PE	180	333	101	34	1	179	DRR079264
	Assembly	Illumina HiSeq 2000	PE	500	277	101	28	1	149	DRR079265
	Scaffolding	Illumina HiSeq 2500	MP	2,500	80	101	8	1	43	DRR079267
	Scaffolding	Illumina HiSeq 2500	MP	3,500	62	101	6	1	33	DRR079268
	Scaffolding	Illumina HiSeq 2500	MP	4,500	62	101	6	1	33	DRR079269
	Scaffolding	Illumina HiSeq 2500	MP	7,000	62	101	6	1	33	DRR079270
	Scaffolding	Illumina HiSeq 2500	MP	11,000	70	101	7	1	37	DRR079271
	Scaffolding	Illumina HiSeq 2500	MP	20,000	261	150	39	1	208	DRR079272
	Gap-closing	Pacific Bioscience RS	SE	6,000	8	1,654‡	11	1	58	DRR079266

RNA-Seq	Gene prediction	Illumina HiSeq 2500	PE	300	249	101	25	1	1,201	DRR419071–DRR419084
	Gene expression analysis	Illumina HiSeq 2500	SE	NA[†]	220	101	22	4×3	89	DRR419085–DRR419096
RAD-Seq	Linkage analysis	Illumina HiSeq 2000	SE	NA[†]	758	101	77	134	90	DRR361370–DRR361503
Re-Seq	Variant calling	Illumina HiSeq 1500	PE	450	448	101	45	12	20	DRR424253–DRR424264
	Variant callling	Illumina HiSeq X Ten	PE	550	1,868	151	282	32	47	DRR424265–DRR424296

[†]NA, not applicable.

[‡]Mean max subread length.

Table S2. Properties of the assembled genome sequence of *Carabus blaptoides fortunei*.

No. of scaffolds	42,502
Scaffold N50, bp	2,655,912
Longest scaffold, bp	7,641,457
Scaffold L50	24
Total length, bp	187,946,950
No. of protein coding genes	16,189
BUSCO scores (for 2,124 orthologues of Endopterygota)	
Complete BUSCOs	2094 (98.6%)
Single-copy	2078 (97.8%)
Duplicated	16 (0.8%)
Fragmented	12 (0.6%)
Missing	18 (0.8%)

Chromosome	No. of genes	No. of *Drosophila* homologs	Locus tag
1	1,005	603	CBL_00001–CBL_01005
2	1,516	943	CBL_01006–CBL_02521
3	1,091	625	CBL_02522–CBL_03612
4	874	573	CBL_03613–CBL_04486

5	1,111	529	CBL_04487–CBL_05597
6	1,299	870	CBL_05598–CBL_06896
7	964	566	CBL_06897–CBL_07860
8	1,090	572	CBL_07861–CBL_08950
9	818	498	CBL_08951–CBL_09768
10	565	338	CBL_09769–CBL_10333
11	763	315	CBL_10334–CBL_11096
12	925	574	CBL_11097–CBL_12021
13	781	454	CBL_12022–CBL_12802
X	1,864	1,275	CBL_12803–CBL_14666
NA*	1,523	459	CBL_20001–CBL_21523
Total	16,189	9,194	

*NA, not assigned on the chromosomes.

Table S3. List of genes located in the body-shape quantitative trait loci region (within the credible interval) that were differentially expressed in the prepupal stages (second instar larvae) of *Carabus blaptoides* subspecies *fortunei* and *capito*. The fragments per kilobase of exon per million reads mapped (FPKM) were calculated, and differential expression was evaluated based on the false discovery rate (FDR)-adjusted *p* value. The location is the base coordinate in scaffold 2061.2.

Location		Blast against *Drosophila*		Head			Thorax		
Start	End	Refseq ID	Gene symbol	*C. capito* FPKM	*C. fortunei* FPKM	FDR-adjusted *p* value	*C. capito* FPKM	*C. fortunei* FPKM	FDR-adjusted *p* value
885,561	890,366	NP_536736.2	*DNApol-alpha180*	8.466	11.857	0.020	5.464	8.295	0.013
897,741	904,102	NA*	NA*	2.439	4.372	0.002	1.874	3.245	0.014
1,001,125	1,003,489	NP_001261949.1	*TMS1*	327.806	207.144	0.002	266.011	175.908	0.005
1,004,058	1,005,642	NP_648182.1	*Uxs*	54.034	31.181	< 0.001	47.207	29.848	0.003
1,006,215	1,007,634	NP_649653.1	*godzilla*	106.781	77.545	0.022	118.152	86.634	0.040
1,040,615	1,057,274	NP_524228.2	*opa*	5.099	10.041	0.006	4.421	7.634	0.042

1,099,314	1,100,859	NP_511074.1	*bys*	37.260	60.192	< 0.001	35.346	59.530	< 0.001
1,101,762	1,102,675	NP_611871.2	*CG11388*	4.030	14.750	< 0.001	1.993	10.610	< 0.001
1,119,685	1,123,673	NP_569983.1	*CG3630*	73.388	424.557	< 0.001	40.103	197.472	< 0.001
1,175,314	1,176,204	NA*	NA†	471.833	648.668	0.023	388.168	557.503	0.019
1,189,178	1,191,738	NP_730693.1	*Nopp140*	112.989	227.463	< 0.001	103.854	208.267	< 0.001
1,197,197	1,197,944	NP_001097577.1	*Adi1*	28.935	44.833	0.032	51.159	76.898	0.022
1,198,157	1,199,098	NP_477419.1	*RpII33*	30.754	46.672	0.008	29.117	41.714	0.044
1,215,444	1,216,032	NA*	NA*	16.691	6.172	0.007	18.006	6.962	0.013
1,220,867	1,223,125	NP_523451.2	*ast*	7.092	10.974	0.013	6.575	10.189	0.027
1,223,502	1,226,911	NP_523466.2	*Ptpa*	28.783	41.648	0.014	25.050	36.489	0.029
1,260,504	1,261,484	NA*	NA*	41.186	84.749	< 0.001	37.932	68.339	< 0.001
1,262,492	1,263,981	NP_610931.1	*O-fut1*	32.341	18.853	< 0.001	28.171	18.552	0.018
1,264,490	1,267,735	NP_611642.2	*GM130*	18.171	35.606	< 0.001	15.183	31.267	< 0.001
1,270,811	1,273,845	NP_001036684.1	*CRAT*	31.564	44.310	0.015	29.289	45.671	0.002
1,275,160	1,278,018	NP_609413.1	*CG7456*	11.498	27.814	< 0.001	9.715	25.030	< 0.001
1,280,010	1,281,955	NP_609710.1	*l(2)34Fc*	6.572	17.311	< 0.001	4.022	10.600	0.006

1,326,831	1,329,761	NP_524025.2	*rt*	10.432	16.531	0.001	9.248	16.078	0.001

*NA, not annotated with a RefSeq protein.

Table S4. Data for individuals following RNA interference (RNAi) treatment.

ID	RNAi gene	Reagent concentration (μg/μL)	Sex	Adult eclosion	Head width	Head length	Thorax width	Thorax length
2204	GFP	1.00	Female	Normal	4.3	10.4	6.9	8.7
2205	GFP	1.00	Female	Normal	4.0	9.5	6.8	8.3
2206	GFP	1.00	Male	Normal	3.7	8.9	6.1	7.8
2207	GFP	1.00	Male	Normal	3.7	9.5	6.6	7.9
2208	GFP	1.00	Female	Normal	4.6	10.2	7.7	9.0
2209	GFP	1.00	Male	Normal	3.4	7.6	5.9	6.4
2210	GFP	1.00	Male	Normal	3.6	9.5	6.2	7.9
2211	GFP	1.00	Female	Normal	4.0	9.8	7.3	8.7
2212	GFP	1.00	Male	Normal	3.6	9.2	6.3	8.2
2213	GFP	1.00	Male	Normal	3.1	7.3	5.9	7.3
2214	GFP	1.00	Female	Normal	3.8	9.6	6.5	8.3
2215	GFP	1.00	Male	Normal	3.7	8.5	6.6	7.8

2216	GFP	1.00	Female	Normal	4.0	9.9	6.9	8.2
2217	GFP	1.00	Female	Normal	4.2	9.4	6.9	8.5
2218	GFP	1.00	Female	Normal	4.1	9.7	7.1	8.3
2219	GFP	1.00	Male	Normal	3.4	8.4	6.0	7.7
2220	GFP	1.00	Female	Normal	4.2	9.8	7.1	9.1
2221	GFP	1.00	Male	Normal	3.6	9.0	6.1	8.0
2222	GFP	1.00	Female	Normal	4.1	8.9	6.7	8.3
2223	GFP	1.00	Male	Normal	3.7	8.9	6.5	8.0
2224	opa	0.10	Female	With defects	3.7	8.7	6.4	6.3
2225	opa	0.10	Unknown	Died in prepupal stage	-	-	-	-
2226	opa	0.10	Female	With defects	3.7	9.0	6.2	6.2
2227	opa	0.10	Female	With defects	3.5	8.6	6.3	6.0
2228	opa	0.10	Unknown	Died in prepupal stage	-	-	-	-
2229	opa	0.10	Male	With defects	3.5	8.1	6.3	6.2
2230	opa	0.10	Female	With defects	3.5	9.2	6.7	6.4
2231	opa	0.10	Female	With defects	3.9	8.4	6.9	7.6

2232	opa	0.10	Unknown	Died in prepupal stage	-	-	-	-
2233	opa	0.10	Male	With defects	3.3	7.6	5.7	5.9
2234	opa	0.10	Female	With defects	3.3	7.8	6.1	5.7
2235	opa	0.10	Unknown	Died in prepupal stage	-	-	-	-
2236	opa	0.10	Male	With defects	3.2	7.9	5.8	6.1
2237	opa	0.10	Female	With defects	4.1	9.4	7.4	7.4
2238	opa	0.10	Unknown	Died in prepupal stage	-	-	-	-
2239	opa	0.10	Unknown	Died in pupal stage	-	-	-	-
2240	opa	0.10	Male	With defects	3.2	7.8	5.6	5.8
2241	opa	0.10	Male	With defects	3.5	8.0	5.9	6.0
2243	opa	0.10	Female	With defects	4.2	9.0	6.4	6.3
2244	opa	0.10	Male	With defects	3.1	7.2	5.7	5.9
2256	opa	0.27	Female	With defects	3.6	8.4	6.3	6.3
2258	opa	0.27	Unknown	Died in prepupal stage	-	-	-	-
2259	opa	0.27	Male	With defects	3.2	7.2	5.9	5.6
2262	opa	0.27	Female	With defects	3.5	8.0	6.0	6.1

2163	opa	0.74	Unknown	Died in prepupal stage	-	-	-	-
2165	opa	0.74	Unknown	Died in prepupal stage	-	-	-	-
2166	opa	0.74	Unknown	Died in prepupal stage	-	-	-	-
2167	opa	0.74	Male	With defects	3.6	7.7	6.1	6.1
2168	opa	0.74	Unknown	Died in prepupal stage	-	-	-	-
2169	opa	0.74	Female	With defects	3.7	8.4	6.8	6.7
2170	opa	0.74	Unknown	Died in prepupal stage	-	-	-	-
2171	opa	0.74	Unknown	Died in prepupal stage	-	-	-	-
2172	opa	0.74	Male	With defects	3.6	8.4	6.0	6.0
2174	opa	0.74	Unknown	Died in prepupal stage	-	-	-	-
2176	opa	0.74	Female	Died in pupal stage	-	-	-	-
2178	opa	0.74	Male	With defects	3.5	7.7	6.4	5.7
2179	opa	0.74	Unknown	Died in prepupal stage	-	-	-	-
2181	opa	0.74	Unknown	Died in prepupal stage	-	-	-	-
2250	opa	0.74	Unknown	Died in prepupal stage	-	-	-	-
2251	opa	0.74	Female	With defects	3.9	8.3	6.5	6.8

2252	opa	0.74	Male	With defects	3.2	7.7	5.8	5.9
2246	opa	0.74	Unknown	Died in prepupal stage	-	-	-	-
2248	opa	0.74	Unknown	Died in prepupal stage	-	-	-	-
2249	opa	0.74	Unknown	Died in prepupal stage	-	-	-	-
2191	opa	2.00	Male	With defects	3.6	7.8	6.2	5.8
2194	opa	2.00	Unknown	Died in prepupal stage	-	-	-	-
2195	opa	2.00	Unknown	Died in pupal stage	-	-	-	-
2196	opa	2.00	Female	With defects	3.6	8.7	6.2	6.4
2197	opa	2.00	Unknown	Died in pupal stage	-	-	-	-
2198	opa	2.00	Unknown	Died in prepupal stage	-	-	-	-
2199	opa	2.00	Female	With defects	3.3	7.9	6.0	5.6
2200	opa	2.00	Female	With defects	3.6	8.4	6.1	6.4
2201	opa	2.00	Male	With defects	3.2	7.3	6.0	5.7
2202	opa	2.00	Unknown	Died in prepupal stage	-	-	-	-
2203	opa	2.00	Unknown	Died in prepupal stage	-	-	-	-
2254	opa	2.00	Female	With defects	3.5	8.0	5.9	5.6

2255	opa	2.00	Female	With defects	4.0	8.9	7.3	6.7
2182	opa	2.00	Unknown	Died in prepupal stage	-	-	-	-
2183	opa	2.00	Unknown	Died in prepupal stage	-	-	-	-
2184	opa	2.00	Male	With defects	3.4	7.9	5.8	5.8
2186	opa	2.00	Unknown	Died in prepupal stage	-	-	-	-
2188	opa	2.00	Unknown	Died in prepupal stage	-	-	-	-
2189	opa	2.00	Male	With defects	3.4	7.5	6.0	5.9
2193	opa	2.00	Unknown	Died in prepupal stage	-	-	-	-

Table S5. Annotation of predicted gene loci within the body size quantitative trait loci (QTL) region of scaffold 52 for *Carabus japonicus*. Results of gene annotation for predicted gene loci within the body size QTL region of scaffold 52 (linkage group 5) in *Carabus japonicus* (modified from Komurai et al. (2017)). RAD loci were used for QTL mapping. GM* indicates the RAD locus at the peak QTL position in GM. Candidate genes are described in Fig. 6 and Table S3 in Komurai et al. (2017) with the addition of *opa* (encoding the zinc finger protein ZIC). Body size measurements: GM, geometric mean of body dimensions; BL, body length; EL, elytral length; TW, thorax width.

QTL region / RAD locus (grey)	Candidate gene	Position start	stop	strand	Gene annotation
BL/EL/TW		261,256			
BL/EL/TW		281,851	292,197	+	gi|195377238|ref|XP_002047399.1| GJ13415 [Drosophila virilis]
BL/EL/TW		309,303	339,724	+	gi|642923391|ref|XP_008193728.1| PREDICTED: protein still life, isoform SIF type 1 [Tribolium castaneum]
BL/EL/TW		339,894	341,356	-	gi|350409592|ref|XP_003488786.1| PREDICTED: growth hormone-regulated TBC protein 1-A-like [Bombus impatiens]
BL/EL/TW		342,149	346,290	+	gi|380025547|ref|XP_003696532.1| PREDICTED: importin-5 [Apis florea]

<table>
<tr><td>BL/EL/TW</td><td></td><td>346,908</td><td>350,020</td><td>-</td><td>gi|642924819|ref|XP_008194054.1| PREDICTED: pre-mRNA 3’-end-processing factor FIP1 isoform X1 [Tribolium castaneum]</td></tr>
<tr><td>BL/EL/TW</td><td></td><td>352,900</td><td>354,740</td><td>-</td><td>gi|642922996|ref|XP_008200488.1| PREDICTED: GATA-binding factor A isoform X2 [Tribolium castaneum]</td></tr>
<tr><td>BL/EL/TW</td><td></td><td>382,810</td><td>414,327</td><td>-</td><td>gi|642923014|ref|XP_008200496.1| PREDICTED: transcription factor GATA-5-like isoform X3 [Tribolium castaneum]</td></tr>
<tr><td>BL/EL/TW</td><td>E2F transcription factor 1 (E2f1)</td><td>426,984</td><td>429,521</td><td>-</td><td>gi|642939444|ref|XP_008200394.1| PREDICTED: transcription factor E2F2-like [Tribolium castaneum]
>gi|642939446|ref|XP_008200396.1| PREDICTED: transcription factor E2F2-like [Tribolium castaneum]
>gi|642939448|ref|XP_008200397.1| PREDICTED: transcription factor E2F2-like [Tribolium castaneum]
>gi|642939450|ref|XP_008200398.1| PREDICTED: transcription factor E2F2-like [Tribolium castaneum]</td></tr>
<tr><td>BL/EL/TW</td><td>pumilio (pum)</td><td>435,892</td><td>439,392</td><td>-</td><td>gi|642939442|ref|XP_008200393.1| PREDICTED: maternal protein pumilio isoform X6 [Tribolium castaneum]</td></tr>
</table>

BL/EL/TW		446,383	447,722	+	NA
BL/EL/TW		455,637	455,951	+	NA
BL/EL/TW	*pumilio* (*pum*)	464,869	473,330	-	NA
BL/EL/TW		475,393	476,608	+	NA
BL/EL/TW	*pumilio* (*pum*)	500,082	502,887	-	gi|642939442|ref|XP_008200393.1| PREDICTED: maternal protein pumilio isoform X6 [Tribolium castaneum]
GM/BL/EL/TW		500,113			
GM/BL/EL/TW		505,147	506,375	+	NA
GM/BL/EL/TW		509,933	512,806	+	NA
GM/BL/EL/TW		519,206	527,858	-	NA
GM/BL/EL/TW		530,542	531,015	+	NA
GM/BL/EL/TW		531,544	532,404	-	gi|642939434|ref|XP_008200389.1| PREDICTED: pumilio homolog 2 isoform X2 [Tribolium castaneum]
GM/BL/EL/TW		538,907	541,401	-	gi|112984034|ref|NP_001037263.1| ribosomal protein S8 [Bombyx mori]
GM/BL/EL/TW		542,193	549,844	-	gi|642921739|ref|XP_008199307.1| PREDICTED: protein kinase

					C-binding protein 1 isoform X1 [Tribolium castaneum] >gi|642921741|ref|XP_008199308.1| PREDICTED: protein kinase C-binding protein 1 isoform X1 [Tribolium castaneum]
GM/BL/EL/TW		551,425	570,205	+	gi|642917156|ref|XP_008191141.1| PREDICTED: filamin-A [Tribolium castaneum]
GM/BL/EL/TW		557,342			
GM/BL/EL/TW		574,752	576,425	-	gi|91079382|ref|XP_971330.1| PREDICTED: zinc finger protein 396 [Tribolium castaneum] >gi|642917154|ref|XP_008191140.1| PREDICTED: zinc finger protein 396 [Tribolium castaneum]
GM/BL/EL/TW		583,441	586,497	-	NA
GM/BL/EL/TW		605,128	606,107	+	gi|347971649|ref|XP_313569.5| AGAP004295-PA [Anopheles gambiae str. PEST]
GM/BL/EL/TW	*CG4063 gene product from transcript CG4063-RA*	606,806	608,367	-	gi|193627197|ref|XP_001950365.1| PREDICTED: F-box-like/WD repeat-containing protein TBL1XR1 [Acyrthosiphon pisum]

	(*ebi*)				
GM/BL/EL/TW		609,236	659,905	+	gi|642921861|ref|XP_008192921.1| PREDICTED: diacylglycerol kinase eta [Tribolium castaneum]
GM/BL/EL/TW		664,294	665,289	+	gi|642921863|ref|XP_008192922.1| PREDICTED: DCN1-like protein 2 isoform X1 [Tribolium castaneum] >gi|642921865|ref|XP_008192924.1| PREDICTED: DCN1-like protein 2 isoform X2 [Tribolium castaneum]
GM/BL/EL/TW	*Ribosomal protein L8* (*RpL8*)	668,346	670,827	+	gi|383847717|ref|XP_003699499.1| PREDICTED: 60S ribosomal protein L8-like [Megachile rotundata]
GM/BL/EL/TW		671,980	674,966	+	gi|237681149|ref|NP_001153721.1| ATP-dependent RNA helicase belle [Tribolium castaneum]
GM/BL/EL/TW		677,178	681,965	+	gi|512892731|ref|XP_004923004.1| PREDICTED: DNA polymerase alpha catalytic subunit-like [Bombyx mori]
GM/BL/EL/TW		682,307	685,658	+	gi|91083351|ref|XP_975052.1| PREDICTED: gamma-tubulin complex component 3 [Tribolium castaneum]

GM/BL/EL/TW	686,805	688,669	-	gi|642923787|ref|XP_008193883.1| PREDICTED: uncharacterized protein F13E6.1 [Tribolium castaneum] >gi|642923789|ref|XP_008193884.1| PREDICTED: uncharacterized protein F13E6.1 [Tribolium castaneum]
GM/BL/EL/TW	689,515	690,165	+	gi|642923784|ref|XP_974439.3| PREDICTED: probable 28S ribosomal protein S26, mitochondrial [Tribolium castaneum]
GM/BL/EL/TW	690,201	690,899	-	gi|91083283|ref|XP_974418.1| PREDICTED: pre-rRNA-processing protein esf2 [Tribolium castaneum]
GM/BL/EL/TW	691,134	695,741	-	gi|642923776|ref|XP_008193879.1| PREDICTED: calponin homology domain-containing protein DDB_G0272472-like [Tribolium castaneum]
GM*/BL/EL/TW	694,037			
GM/BL/EL/TW	697,860	733,591	-	gi|642923778|ref|XP_008193880.1| PREDICTED: zinc finger protein 395 isoform X1 [Tribolium castaneum]
GM/BL/EL/TW	741,712	750,582	-	gi|242020223|ref|XP_002430555.1| conserved hypothetical protein [Pediculus humanus corporis]

<table>
<tr><td>GM/BL/EL/TW</td><td></td><td>752,995</td><td>760,070</td><td>-</td><td>gi|642923782|ref|XP_008193882.1| PREDICTED: regulating synaptic membrane exocytosis protein 2 [Tribolium castaneum]</td></tr>
<tr><td>GM/BL/EL/TW</td><td>brother of tout-velu (botv)</td><td>760,279</td><td>764,825</td><td>-</td><td>gi|91083291|ref|XP_974527.1| PREDICTED: exostosin-3 [Tribolium castaneum]</td></tr>
<tr><td>GM/BL/EL/TW</td><td></td><td>765,244</td><td>766,024</td><td>-</td><td>gi|340712465|ref|XP_003394780.1| PREDICTED: nuclear cap-binding protein subunit 2-like [Bombus terrestris] >gi|350399792|ref|XP_003485640.1| PREDICTED: nuclear cap-binding protein subunit 2-like [Bombus impatiens]</td></tr>
<tr><td>GM/BL/EL/TW</td><td></td><td>766,243</td><td>775,912</td><td>-</td><td>gi|91083281|ref|XP_974400.1| PREDICTED: nose resistant to fluoxetine protein 6 [Tribolium castaneum]</td></tr>
<tr><td>GM/BL/EL/TW</td><td></td><td>783,735</td><td>790,588</td><td>-</td><td>gi|288869514|ref|NP_001165864.1| extended synaptotagmin-like protein 2a [Tribolium castaneum] >gi|642923659|ref|XP_008193831.1| PREDICTED: extended synaptotagmin-like protein 2a isoform X1 [Tribolium castaneum]</td></tr>
<tr><td>GM/BL/EL/TW</td><td></td><td>795,388</td><td>797,778</td><td>-</td><td>gi|642923926|ref|XP_008193930.1| PREDICTED: probable serine incorporator isoform X3 [Tribolium castaneum]</td></tr>
</table>

GM/BL/EL/TW		798,347	799,770	-	gi|157112666|ref|XP_001651838.1| dtdp-glucose 4-6-dehydratase [Aedes aegypti]
GM/BL/EL/TW		800,638	802,051	+	gi|380017760|ref|XP_003692814.1| PREDICTED: E3 ubiquitin-protein ligase RNF13-like [Apis florea]
GM/BL/EL/TW		803,124	810,649	-	gi|512918195|ref|XP_004929073.1| PREDICTED: pickpocket protein 28-like [Bombyx mori]
GM/BL/EL/TW		816,218	818,919	+	NA
GM/BL/EL/TW	*zinc finger protein ZIC* = *odd paired* (*opa*)	839,510	841,144	+	gi|242013026|ref|XP_002427222.1| zinc finger protein ZIC, putative [Pediculus humanus corporis]
GM/BL/EL/TW	*zinc finger protein ZIC* = *odd paired* (*opa*)	854,325	855,866	+	gi|242013026|ref|XP_002427222.1| zinc finger protein ZIC, putative [Pediculus humanus corporis]
GM/BL/EL/TW		859,723	860,025	+	NA
GM/BL/EL/TW		869,632	870,939	-	NA
GM/BL/EL/TW		883,822			

Table S6. Phenotypic values used in the morphometric analysis of *C. b. capito* and *C. b. fortunei*.

ID	Subspecies	Sex	Head width	Head length	Thorax width	Thorax length
C20001	*C. b. capito*	Female	4.63	9.25	6.41	7.02
C20002	*C. b. capito*	Male	4.23	8.48	6.62	7.06
C20003	*C. b. capito*	Female	5.03	9.46	7.32	7.79
C20004	*C. b. capito*	Female	4.75	8.99	6.95	7.07
C20005	*C. b. capito*	Male	3.87	7.45	5.55	6.21
C20006	*C. b. capito*	Male	4.47	8.74	6.79	7.11
C20007	*C. b. capito*	Male	4.07	8.1	6.44	6.05
C20008	*C. b. capito*	Male	4.22	8.31	6.68	6.84
C20009	*C. b. capito*	Female	5.2	8.95	6.93	6.93
C20010	*C. b. capito*	Female	5.21	9.96	7.64	7.77
C20012	*C. b. capito*	Male	4.19	8.65	6.37	6.52
C20013	*C. b. capito*	Female	4.81	9.25	6.97	7.44
C20014	*C. b. capito*	Male	4.16	8.14	6.6	6.69
C20015	*C. b. capito*	Female	5.27	9.71	7.64	7.65
C20016	*C. b. capito*	Male	4.23	8.37	6.79	6.95
C20019	*C. b. capito*	Female	5.02	10.05	7.5	7.96
F20001	*C. b. fortunei*	Female	3.87	8.86	6.03	8.13
F20003	*C. b. fortunei*	Female	4.65	9.31	6.57	8.51
F20004	*C. b. fortunei*	Female	3.57	7.67	6.12	6.88
F20005	*C. b. fortunei*	Female	4.27	9.81	7.39	8.63
F20006	*C. b. fortunei*	Female	3.88	9.33	6.48	8.05

F20007	*C. b. fortunei*	Male	3.49	7.99	5.74	7.65
F20008	*C. b. fortunei*	Male	3.63	8.81	6.18	7.69
F20009	*C. b. fortunei*	Female	3.98	9.26	6.62	8.54
F20010	*C. b. fortunei*	Female	4.34	9.73	7.18	8.79
F20011	*C. b. fortunei*	Male	3.68	8.79	6.56	7.98
F20012	*C. b. fortunei*	Female	3.63	8.03	6.39	7.24
F20013	*C. b. fortunei*	Male	3.73	8.98	6.44	7.99
F20014	*C. b. fortunei*	Male	3.8	9.14	6.66	8.25
F20016	*C. b. fortunei*	Male	3.37	7.63	6.38	7.42
F20018	*C. b. fortunei*	Male	3.49	7.81	6.03	6.83
F20019	*C. b. fortunei*	Male	3.73	8.77	6.49	7.79

Table S7. Phenotypic values used in the quantitative trait locus (QTL) mapping.

ID	Sex	Head width	Head length	Thorax width	Thorax length
1711	Male	3.66	7.89	6.04	6.95
1714	Male	3.80	8.44	6.33	7.11
1716	Male	3.86	8.52	6.59	7.10
1719	Male	3.92	8.66	6.77	7.25
G21001	Female	4.56	9.43	6.96	7.72
G21002	Male	3.72	7.98	6.22	6.93
G21003	Female	4.31	9.12	6.85	7.50
G21004	Female	4.49	9.14	6.90	7.53
G21005	Male	3.80	8.20	6.67	7.18
G21006	Female	4.23	9.40	7.22	7.53
G21007	Male	3.88	8.47	6.40	7.16
G21009	Male	3.94	8.18	6.55	6.89
G21010	Female	4.47	9.31	7.10	7.74
G21011	Female	3.93	8.65	6.83	7.34
G21012	Female	4.29	9.20	7.12	7.72
G21013	Female	3.74	7.93	6.37	6.37
G21014	Female	4.25	9.32	6.83	7.70
G21015	Female	4.29	9.18	7.19	7.78
G21016	Female	4.40	9.07	7.45	8.24
G21017	Female	4.09	8.56	6.66	7.47
G21018	Male	3.80	8.56	6.53	7.20
G21019	Female	4.22	8.80	6.50	7.47
G21020	Female	3.91	8.14	6.33	6.83

G21021	Male	4.15	9.00	7.14	7.70
G21022	Female	4.68	9.70	7.18	8.27
G21023	Female	4.16	8.95	6.68	7.56
G21024	Male	3.77	8.36	6.47	6.95
G21025	Male	3.71	8.03	6.28	6.81
G21026	Male	3.85	8.29	6.56	7.18
G21027	Female	4.87	10.18	7.56	8.54
G21028	Female	4.11	8.51	6.56	7.48
G21030	Male	3.88	8.56	6.54	7.48
G21031	Male	4.06	8.72	6.64	7.36
G21032	Male	3.82	8.40	6.39	6.91
G21033	Male	3.88	8.47	6.72	7.20
G21034	Male	3.92	8.29	6.58	6.92
G21035	Male	4.14	8.75	7.22	7.14
G21036	Female	3.51	7.69	5.99	6.40
G21037	Female	3.86	8.31	6.57	6.78
G21038	Female	4.77	10.08	7.47	8.15
G21039	Male	3.66	8.35	6.33	7.17
G21040	Female	4.22	8.88	6.91	7.59
G21041	Male	3.51	7.65	5.87	6.68
G21042	Female	4.11	9.03	6.94	7.67
G21043	Female	4.38	8.99	6.66	7.47
G21044	Male	4.14	8.77	6.93	7.56
G21045	Female	4.10	8.75	6.43	7.18
G21046	Male	3.94	8.54	6.68	7.12

G21047	Male	3.64	7.94	6.07	6.99
G21048	Female	4.46	9.33	7.25	7.93
G21049	Male	3.76	8.06	6.41	7.28
G21050	Male	3.84	8.49	6.56	7.36
G21051	Female	4.32	9.44	7.26	7.86
G21052	Female	4.53	9.59	7.48	8.30
G21053	Female	4.40	9.49	6.97	7.97
G21054	Male	4.03	8.41	6.43	6.99
G21055	Female	4.05	8.78	5.99	7.21
G21056	Female	4.37	9.20	7.37	7.67
G21057	Male	3.39	7.26	5.40	5.74
G21058	Male	3.89	8.53	6.60	7.08
G21059	Male	3.91	8.62	6.63	7.13
G21060	Male	4.02	8.28	6.57	6.99
G21061	Female	4.33	9.07	7.08	7.54
G21062	Male	3.94	8.46	6.72	6.93
G21063	Male	4.08	8.89	6.62	7.20
G21064	Female	4.60	9.81	7.12	7.84
G21065	Female	4.11	9.21	7.31	7.53
G21066	Male	3.98	8.47	6.52	7.13
G21067	Female	4.10	8.81	6.46	7.25
G21068	Female	4.54	9.60	7.41	8.09
G21069	Male	3.89	8.19	6.46	6.97
G21070	Male	3.93	8.62	6.42	7.26
G21071	Female	3.97	8.48	6.62	6.66

G21072	Female	4.60	9.66	7.44	8.12
G21073	Male	3.68	8.32	6.60	7.08
G21074	Female	4.60	9.41	7.29	7.90
G21075	Female	4.19	8.92	6.43	7.39
G21076	Male	3.23	7.33	5.62	6.32
G21077	Male	3.63	7.50	5.79	6.20
G21078	Female	3.82	8.18	6.03	6.63
G21079	Male	3.79	7.82	6.00	6.53
G21080	Male	3.73	8.07	6.21	6.80

Table S8. Samples used in whole genome resequencing.

ID	Subspecies	Sex	Sampling date	Location	Platform
01	*C. b. capito*	Male	16/5/2012	Sado Island	Illumina HiSeq 1500
02	*C. b. capito*	Male	16/5/2012	Sado Island	Illumina HiSeq 1500
03	*C. b. capito*	Male	16/5/2012	Sado Island	Illumina HiSeq 1500
04	*C. b. capito*	Male	16/5/2012	Sado Island	Illumina HiSeq 1500
05	*C. b. capito*	Male	16/5/2012	Sado Island	Illumina HiSeq 1500
06	*C. b. capito*	Male	16/5/2012	Sado Island	Illumina HiSeq 1500
07	*C. b. fortunei*	Male	25/6/2014	Awashima Island	Illumina HiSeq 1500
08	*C. b. fortunei*	Male	25/6/2014	Awashima Island	Illumina HiSeq 1500
09	*C. b. fortunei*	Male	25/6/2014	Awashima Island	Illumina HiSeq 1500
10	*C. b. fortunei*	Male	25/6/2014	Awashima Island	Illumina HiSeq 1500
11	*C. b. fortunei*	Male	25/6/2014	Awashima Island	Illumina HiSeq 1500
12	*C. b. fortunei*	Male	25/6/2014	Awashima Island	Illumina HiSeq 1500
13	*C. b. capito*	Female	1/6/2016	Sado Island	Illumina HiSeq X Ten
14	*C. b. capito*	Female	9/8/2017	Sado Island	Illumina HiSeq X Ten
15	*C. b. capito*	Male	27/8/2018	Sado Island	Illumina HiSeq X Ten
16	*C. b. capito*	Male	8/8/2017	Sado Island	Illumina HiSeq X Ten
17	*C. b. capito*	Male	16/5/2012	Sado Island	Illumina HiSeq X Ten
18	*C. b. capito*	Male	16/5/2012	Sado Island	Illumina HiSeq X Ten
19	*C. b. capito*	Male	16/5/2012	Sado Island	Illumina HiSeq X Ten
20	*C. b. capito*	Male	16/5/2012	Sado Island	Illumina HiSeq X Ten

21	*C. b. fortunei*	Female	25/6/2014	Awashima Island	Illumina HiSeq X Ten
22	*C. b. fortunei*	Female	25/6/2014	Awashima Island	Illumina HiSeq X Ten
23	*C. b. fortunei*	Male	25/6/2014	Awashima Island	Illumina HiSeq X Ten
24	*C. b. fortunei*	Male	25/6/2014	Awashima Island	Illumina HiSeq X Ten
25	*C. b. fortunei*	Male	25/6/2014	Awashima Island	Illumina HiSeq X Ten
26	*C. b. fortunei*	Male	12/10/2016	Awashima Island	Illumina HiSeq X Ten
27	*C. b. fortunei*	Male	15/5/2017	Awashima Island	Illumina HiSeq X Ten
28	*C. b. fortunei*	Male	15/5/2017	Awashima Island	Illumina HiSeq X Ten
29	*C. b. capito*	Female	1/3/2018	Sado Island	Illumina HiSeq X Ten
30	*C. b. capito*	Female	1/3/2018	Sado Island	Illumina HiSeq X Ten
31	*C. b. capito*	Female	27/8/2018	Sado Island	Illumina HiSeq X Ten
32	*C. b. capito*	Male	27/8/2018	Sado Island	Illumina HiSeq X Ten
33	*C. b. capito*	Male	27/8/2018	Sado Island	Illumina HiSeq X Ten
34	*C. b. capito*	Male	27/8/2018	Sado Island	Illumina HiSeq X Ten
35	*C. b. capito*	Male	16/5/2012	Sado Island	Illumina HiSeq X Ten
36	*C. b. capito*	Male	16/5/2012	Sado Island	Illumina HiSeq X Ten
37	*C. b. fortunei*	Female	25/6/2014	Awashima Island	Illumina HiSeq X Ten
38	*C. b. fortunei*	Female	25/6/2014	Awashima Island	Illumina HiSeq X Ten
39	*C. b. fortunei*	Female	25/6/2014	Awashima Island	Illumina HiSeq X Ten
40	*C. b. fortunei*	Male	25/6/2014	Awashima Island	Illumina HiSeq X Ten
41	*C. b. fortunei*	Male	12/10/2016	Awashima Island	Illumina HiSeq X Ten
42	*C. b. fortunei*	Male	12/10/2016	Awashima Island	Illumina HiSeq X Ten
43	*C. b. fortunei*	Male	15/5/2017	Awashima Island	Illumina HiSeq X Ten

44	*C. b. fortunei*	Male	15/5/2017	Awashima Island	Illumina HiSeq X Ten

Supplementary references

Akiyama K, Jang TW, Park YH, Shinohara T, Konuma J, Liang H, Kubota K, Sota T, Ishikawa R, Kim JL, et al. 2020. Phylogeographical analysis of character displacement in feeding phenotypes of snail-feeding *Acoptolabrus* ground beetles. *Biol J Linn Soc.* 131:936–951.

Aruga J, Kamiya A, Takahashi H, Fujimi TJ, Shimizu Y, Ohkawa K, Yazawa S, Umesono Y, Noguchi H, Shimizu T, et al. 2006. A wide-range phylogenetic analysis of Zic proteins: implications for correlations between protein structure conservation and body plan complexity. *Genomics* 87:783–792.

DeWitt TJ, Robinson BW, Wilson DS. 2000. Functional diversity among predators of a freshwater snail imposes an adaptive trade-off for shell morphology. *Evol Ecol Res.* 2:129–148.

Hursh DA, Stultz BG. 2018. *Odd-Paired*: the *Drosophila* Zic *gene*. *Adv Exp Med Biol.* 1046, 41–58.

Ishikawa R. 1978. A revision of the higher taxa of the subtribe Carabina (Coleoptera, Carabidae). *Bull Nat Sci Mus Ser. A* 5:95–114.

Komurai R, Fujisawa T, Okuzaki Y, Sota T. 2017. Genomic regions and genes related to inter-population differences in body size in the ground beetle *Carabus japonicus*. *Sci Rep.* 7:7773.

Konuma J, Chiba S. 2007. Trade-offs between force and fit: extreme morphologies associated with feeding behavior in carabid beetles. *Am Nat.* 170:90–100.

Konuma J, Nagata N, Sota T. 2011. Factors determining the direction of ecological specialization in snail-feeding carabid beetles. *Evolution* 65:408–418.

Konuma J, Sota T, Chiba S. 2013. A maladaptive intermediate form: a strong trade-off revealed by hybrids between two forms of a snail-feeding beetle. *Ecology* 94:2638–2644.

Mizugishi K, Hatayama M, Tohmonda T, Ogawa M, Inoue T, Mikoshiba K, Aruga J. 2004. Myogenic repressor I-mfa interferes with the function of Zic family proteins. *Biochem Biophys Res Commun.* 320:233–240.

Sota T. 2022. Evolutionary Biology of Carabus Ground Beetles. Singapore: Springer Singapore

Sturani M. 1962. Osservazioni e ricerche biologiche sul genere Carabus Linnaeus (Sensu lato). *Estratto dalle Mem della Soc Entomol Ital.* 41:85–202.

Vermeij GJ. 1979. Shell architecture and causes of death of Micronesian reef snails. *Evolution* 33:686–696.

Wolfe SA, Nekludova L, Pabo CO. 2000. DNA recognition by Cys_2 His_2 zinc finger proteins. *Annu Rev Biophys Biomol. Struct.* 29:183–212.
